# Nuclear Calcium Signaling Controls Expression of a Large Gene Pool: Identification of a Gene Program for Acquired Neuroprotection Induced by Synaptic Activity

**DOI:** 10.1371/journal.pgen.1000604

**Published:** 2009-08-14

**Authors:** Sheng-Jia Zhang, Ming Zou, Li Lu, David Lau, Désirée A. W. Ditzel, Celine Delucinge-Vivier, Yoshinori Aso, Patrick Descombes, Hilmar Bading

**Affiliations:** 1 Department of Neurobiology, Interdisciplinary Center for Neurosciences (IZN), University of Heidelberg, Heidelberg, Germany; 2 Genomics Platform, Centre Médical Universitaire, Université de Genève, Geneva, Switzerland; University of Minnesota, United States of America

## Abstract

Synaptic activity can boost neuroprotection through a mechanism that requires synapse-to-nucleus communication and calcium signals in the cell nucleus. Here we show that in hippocampal neurons nuclear calcium is one of the most potent signals in neuronal gene expression. The induction or repression of 185 neuronal activity-regulated genes is dependent upon nuclear calcium signaling. The nuclear calcium-regulated gene pool contains a genomic program that mediates synaptic activity-induced, acquired neuroprotection. The core set of neuroprotective genes consists of 9 principal components, termed *Activity-regulated Inhibitor of Death* (*AID*) genes, and includes *Atf3*, *Btg2*, *GADD45β*, *GADD45γ*, *Inhibin β-A*, *Interferon activated gene 202B*, *Npas4*, *Nr4a1*, and *Serpinb2*, which strongly promote survival of cultured hippocampal neurons. Several *AID* genes provide neuroprotection through a common process that renders mitochondria more resistant to cellular stress and toxic insults. Stereotaxic delivery of *AID* gene-expressing recombinant adeno-associated viruses to the hippocampus confers protection *in vivo* against seizure-induced brain damage. Thus, treatments that enhance nuclear calcium signaling or supplement *AID* genes represent novel therapies to combat neurodegenerative conditions and neuronal cell loss caused by synaptic dysfunction, which may be accompanied by a deregulation of calcium signal initiation and/or propagation to the cell nucleus.

## Author Summary

The dialogue between the synapse and the nucleus plays an important role in the physiology of neurons because it links brief changes in the membrane potential to the transcriptional regulation of genes critical for neuronal survival and long-term memory. The propagation of activity-induced calcium signals to the cell nucleus represents a major route for synapse-to-nucleus communication. Here we identified nuclear calcium-regulated genes that are responsible for a neuroprotective shield that neurons build up upon synaptic activity. We found that among the 185 genes controlled by nuclear calcium signaling, a set of 9 genes had strong survival promoting activity both in cell culture and in an animal model of neurodegeneration. The mechanism through which several genes prevent cell death involves the strengthening of mitochondria against cellular stress and toxic insults. The discovery of an activity-induced neuroprotective gene program suggest that impairments of synaptic activity and synapse-to-nucleus signaling, for example due to expression of Alzheimer's disease protein or in aging, may comprise the cells' own neuroprotective system eventually leading to cell death. Thus, malfunctioning of nuclear calcium signaling could be a key etiological factor common to many neuropathological conditions, providing a simple and unifying concept to explain disease- and aging-related cell loss.

## Introduction

Physiological levels of synaptic activity are required for neurons to survive [1]. Activity-dependent neuroprotection is induced by calcium entry through synaptic NMDA receptors and requires that calcium transients invade the cell nucleus [2]–[6]. Procedures that interfere with electrical activity and compromise NMDA receptor function or nuclear calcium signaling can have deleterious effects on the health of neurons both *in vitro* and *in vivo*. For example, blockade of NMDA receptors *in vivo* following intraperitoneal injections of the NMDA receptor antagonist MK-801 into seven day-old rats triggers, within 24 hours, a wave of apoptotic neurodegeneration in many brain regions, including the parietal and frontal cortex, the thalamus and the hippocampus [7]. Likewise, the selective blockade of nuclear calcium signaling prevents cultured hippocampal neurons from building up anti-apoptotic activity upon synaptic NMDA receptor stimulation [2],[3],[6]. Conversely, enhancing neuronal firing and synaptic NMDA receptor activity is neuroprotective: networks of cultured hippocampal neurons that have experienced periods of action potential bursting causing calcium entry through synaptic NMDA receptors are more resistant to cell death-inducing conditions [2]–[4]. Moreover, stimulating synaptic activity *in vivo* by exposing rats to enriched environments reduces spontaneous apoptotic cell death in the hippocampus and protects against neurotoxic injuries [8].

Neuronal activity and NMDA receptor-induced calcium signaling pathways can suppress apoptosis and promote survival through two mechanistically distinct processes. One process is independent of on-going gene transcription and involves the phosphatidylinositide 3′-OH kinase (PI3K)-AKT signaling pathway which promotes survival while neurons are being electrically stimulated [3]. However, the principal pathway conferring long-lasting neuroprotection requires the generation of calcium transients in the cell nucleus [2]–[6],[9]. The aim of this study was to investigate how nuclear calcium promotes neuroprotection. Using tools to selectively block nuclear calcium signaling in hippocampal neurons in conjunction with microarray technologies and bioinformatics, we uncovered a genomic survival program that is induced by calcium transients in the cell nucleus. The core components of this program, referred to as *Activity-regulated Inhibitor of Death (AID)* genes, can provide neurons with a broad-spectrum neuroprotective shield against cell death.

## Results

### Identification of nuclear calcium–regulated genes

**Figure 1:**
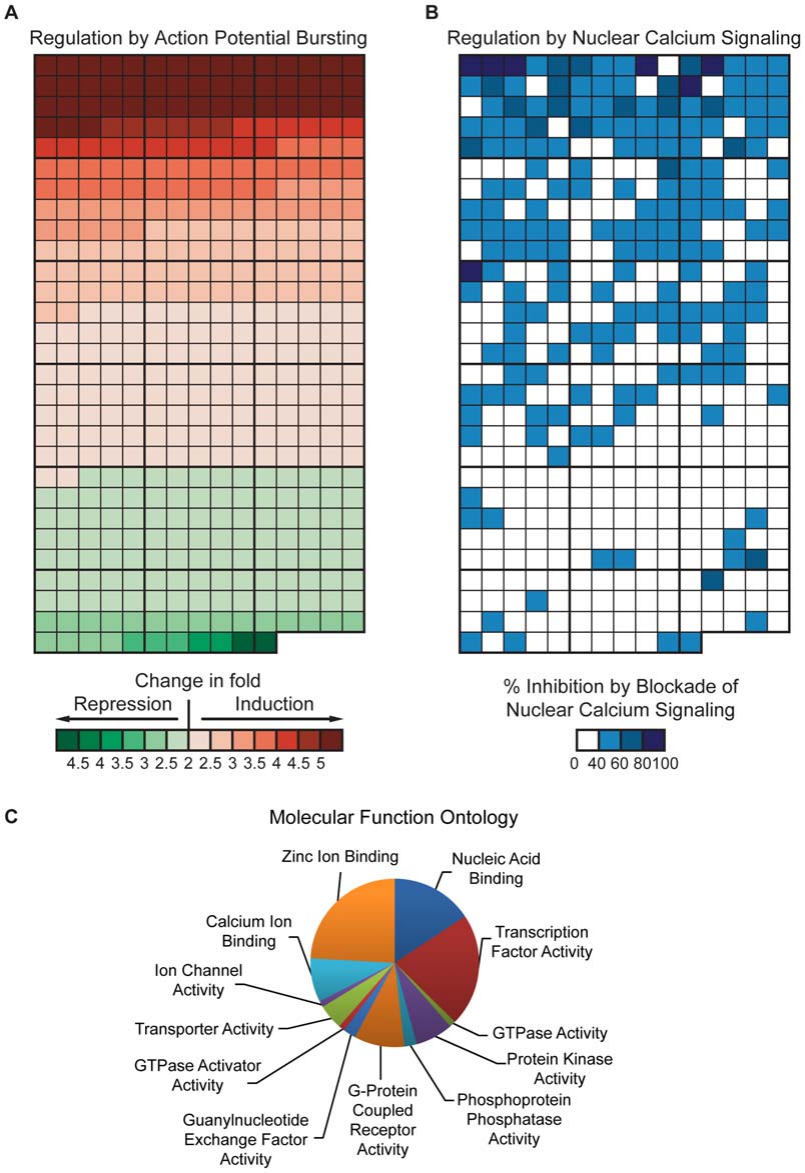
Color-coded map of genes regulated by neuronal activity and nuclear calcium signaling and their molecular functions. Each box corresponds to one gene whose position within the two maps is identical; corresponding positions on the map indicate the regulation of a given gene by AP bursting and nuclear calcium signaling. (A) The maximum fold change in expression (induction or repression) based on Affymetrix microarray analysis at 4 hours after the on-set of action potential (AP) bursting is color-coded as indicated. Genes are sorted on the basis of their induction/repression values in a “descending” order, i.e. from the highest levels of induction following AP bursting at the top (indicated in dark red color) to the highest level of repression following AP bursting at the bottom (indicated in dark green color). (B) Genes whose induction or repression by AP bursting is inhibited by expression of CaMBP4 are highlighted in blue; more than 80% inhibition (dark blue), between 60 and 80% inhibition (light blue), between 40 and 60% inhibition (very light blue). White boxes indicate genes whose induction or repression by AP bursting is inhibited less than 40% by expression of CaMBP4. The complete list of all blue-indicated genes is given in Table 1. (C) Molecular functions of the 185 nuclear calcium-regulated genes based on Gene Ontology information provided by Affymetrix (http://www.affymetrix.com).

To identify genes regulated by nuclear calcium signaling in hippocampal neurons, we carried out comparative whole-genome transcriptional profiling. Hippocampal neurons were infected with a recombinant adeno-associated virus (rAAV) expressing either the calmodulin (CaM) binding-peptide, CaMBP4 (rAAV-*CaMBP4* [6]) or *β-galactosidase* (rAAV-*LacZ*) as a control. CaMBP4 is a nuclear protein that contains 4 repeats of the M13 calmodulin binding peptide derived from the rabbit skeletal muscle myosin light chain kinase; it binds to and inactivates the nuclear calcium/CaM complex [10]. Inhibition of nuclear calcium signaling with CaMBP4 in hippocampal neurons blocks synaptic activity-evoked CREB-mediated transcription and prevents the induction of a genomic neuroprotective program by neuronal activity [3],[6]. Hippocampal neurons were stimulated by exposing the network to the GABA_A_ receptor antagonist, bicuculline. GABAergic interneurons, which represent about 11% of the neuron population, impose a tonic inhibition onto the network [11]. Removal of GABA_A_ergic inhibition with bicuculline leads to action potential (AP) bursting, which stimulates calcium entry though synaptic NMDA receptors, generates robust cytoplasmic and nuclear calcium transients, induces CREB-dependent transcription, and strongly promotes neuronal survival [2]–[4],[6],[11],[12]. RNA isolated from these hippocampal neurons was used for microarray analyses on Affymetrix GeneChips. The Affymetrix microarray data were analyzed by a two-step process; details of the data analysis are described in Text S1. First, we determined all genes induced or repressed by AP bursting (which gives rise to robust nuclear calcium signals) in control-infected (rAAV-*LacZ*) hippocampal neurons. A threshold of 2.0 fold was chosen, which, given that microarray data are compressed and generally underestimates the fold differences in gene expression [6],[13], filters out genes that are likely to undergo signal-induced changes in their expression that are in the range of at least 2.5 to 3 fold. This analysis revealed 302 genes that were induced and 129 genes that were repressed in rAAV-*LacZ* infected hippocampal neurons 4 hours after the induction of AP bursting. A color-coded map provides an overview of these 431 AP bursting-regulated genes (Figure 1A). A comparison of the genes identified in this study using rAAV-*LacZ* infected hippocampal neurons with the pool of activity-regulated genes described in a previous study [6] revealed a high degree of overlap. However, due to the higher threshold applied in this analysis (2 fold vs. 1.5 fold change used in our previous study [6]), the current analysis filtered out fewer genes.

In a second analysis step, we compared the expression of genes regulated by AP bursting in hippocampal neurons infected with rAAV-*LacZ* and in hippocampal neurons infected with rAAV-*CaMBP4* to block the nuclear calcium/CaM complex. The regulation of a gene was considered dependent on nuclear calcium signaling if based on the microarray data its induction or repression by AP bursting is reduced by at least 40% in rAAV-*CaMBP4* infected neurons compared to rAAV-*LacZ* infected neurons. We found that 183 genes (plus *Btg2* and *Bcl6*; see below) fulfill these criteria (Figure 1B); a list of those genes including their fold changes following AP bursting and percent inhibition by CaMBP4 is given in Table 1. We are likely to underestimate the total number of genes regulated by nuclear calcium signaling for several reasons. First, our screen did not identify genes controlled by the downstream regulatory element antagonist modulator (DREAM) [14]. DREAM is a transcriptional repressor that can directly bind calcium through its EF hands. In its calcium-bound form, DREAM is released from the DNA allowing transcription to be activated in a nuclear calcium-dependent but calmodulin-independent manner [14]. Second, our analysis was restricted to one time point (i.e. 4 hours after induction of AP bursting) and therefore it is possible that we have missed genes that have peak expression levels at time points significantly earlier or later than 4 hours. Although those genes may also show changes in expression levels at 4 hours after AP bursting, if this induction was less than two fold they were not scored as induced in our study. Third, a possible regulation by nuclear calcium could also have been missed because for genes weakly induced by AP bursting, the accuracy of the microarray data–based assessment of nuclear calcium regulation decreases and the necessary statistical criteria may not be met. Indeed, these conditions apply to two neuronal survival-promoting genes, *Btg2* and *Bcl6* [6]. *Btg2* is induced with fast kinetics after AP bursting; the induction peaks at 2 hours [about 8 fold induction based on microarray data and 17 fold induction based on quantitative reverse transcriptase (QRT)-PCR analysis [6]], whereas at the 4 hours time point the change in expression based on microarray data relative to unstimulated control is only about 2 fold [6]. In the case of *Bcl6*, the fold changes observed at 4 hours after the on-set of AP bursting are about 1.5 fold based on microarray data analysis and about 2.5 fold based on QRT-PCR analysis [6]. Both, *Btg2* and *Bcl6*, are regulated by nuclear calcium signaling [6] and have therefore been included in the list of nuclear calcium-regulated genes (Table 1). The identified nuclear calcium-regulated gene pool comprises 43% of all activity-regulated genes. It contains a large variety of gene products with different catalytic and binding activities (Figure 1C).

**Table 1:** Genes regulated by nuclear calcium signaling in hippocampal neurons.

ID	Gene Name	ACT/REP	INH	ID	Gene Name	ACT/REP	INH	ID	Gene Name	ACT/REP	INH
AB019028	*Crsp2*	+	*	NM_008416	*Junb*	+++	**	NM_021382	*Tacr3*	+	**
AI507491	*1110020A10Rik*	+	**	NM_008543	*Smad7*	+	*	NM_021458	*Fzd3*	+	*
AI597036		+	*	NM_008548	*Man1a*	+	*	NM_021462	*Mknk2*	+	*
AK004371	*Rasl11a*	++	*	NM_008562	*Mcl1*	+	*	NM_021788	*Sap30*	++	*
AK013051	*2810409C01Rik*	+	*	NM_008565	*Mcm4*	−	**	NM_022995	*Tmepai*	+	*
AK020483	*Malat1*	+	*	NM_008655	*Gadd45β*	+++	**	NM_023324	*Peli1*	+	*
AK021003	*B230216N24Rik*	+	**	NM_008717	*Zfml*	+	*	NM_023503	*Ing2*	+	*
AV309085	*1190002N15Rik*	++	**	NM_008780	*Pax1*	+	*	NM_024166	*2410018M08Rik*	−	**
AV348660	*Gm1568*	−	*	NM_008842	*Pim1*	+++	*	NM_024285	*Bves*	+	*
AW109901	*4933411B09Rik*	+	*	NM_008872	*Plat*	++	*	NM_025404	*Arfl4*	+	*
AW490446	*4930505D03Rik*	−	**	NM_008924	*Prkar2a*	+	*	NM_025413	*Lce1g*	+++	***
AW555393	*Mest*	++	*	NM_008965	*Ptger4*	+	*	NM_025635	*Zwint*	+	*
BB009682	*4930434J08Rik*	+	**	NM_008987	*Ptx3*	++	*	NM_025775	*Tmtc2*	+	**
BB013412		−	*	NM_009013	*Rad51ap1*	−	*	NM_026153	*5730557B15Rik*	++	**
BB096900	*4933402J24Rik*	+	*	NM_009044	*Rel*	++	**	NM_026324	*Kirrel3*	+	**
BB161981		+	*	NM_009230	*Soat1*	+	*	NM_026394	*Lce1f*	+	**
BB194610	*A330084C13Rik*	+	**	NM_009264	*Sprr1a*	+	*	NM_026989	*Sfrs11*	+	*
BB246700	*A630072M18Rik*	+	*	NM_009551	*Zfand5*	+	*	NM_027514	*Pvr*	+	*
BB253137	*Inhbb*	−−	*	NM_009744	*Bcl6*	+	Ref.	NM_027518	*6330416L11Rik*	+	**
BB268139	*Ibrdc1*	+	*	NM_009769	*Klf5*	+	*	NM_027559	*BC063749*	++	*
BB322180	*Phf21b*	+	*	NM_009859	*Sept7*	+	*	NM_028067	*Tsc22d2*	+	*
BB322233	*5430433G21Rik*	++	*	NM_009883	*Cebpb*	+	*	NM_028755	*Arpp21*	+	*
BB354702		−	*	NM_009911	*Cxcr4*	+	**	NM_028760	*Cep55*	++	**
BB387595	*Gprin2*	−	*	NM_010193	*Fem1b*	++	*	NM_028829	*Paqr8*	+	**
BB389395	*Onecut2*	+	*	NM_010207	*Fgfr2*	++	**	NM_029466	*Arl5b*	++	*
BB398124	*C330006P03Rik*	+++	*	NM_010276	*Gem*	+	*	NM_029667	*Lce1i*	++	**
BB464727	*A830010M20Rik*	+	*	NM_010444	*Nr4a1*	+++	*	NM_029688	*Srxn1*	+	*
BB473548		+	*	NM_010500	*Ier5*	+	*	NM_053011	*Lrp1b*	+	**
BB560177	*LOC620695*	+	*	NM_010516	*Cyr61*	+++	**	NM_053182	*Pag1*	+	*
BB667130	*2210038L17Rik*	+	**	NM_011012	*Oprl1*	−	**	NM_080433	*Zfp312*	+	*
BB667296	*Ptprk*	+	**	NM_011111	*Serpinb2*	+++	***	NM_080726	*Rem2*	+	***
BC023116	*Cgref1*	+	*	NM_011198	*Ptgs2*	+++	**	NM_080853	*Slc17a6*	+	*
BE447663	*Heca*	+	*	NM_011245	*Rasgrf1*	+	*	NM_130447	*Dusp16*	+	*
BE686667	*Slco5a1*	+	*	NM_011361	*Sgk*	++	*	NM_133236	*Glcci1*	+	*
BE687858	*BC023892*	++	*	NM_011401	*Slc2a3*	+	*	NM_133753	*Errfi1*	++	*
BE691546	*C030046G05*	++	**	NM_011607	*Tnc*	+	*	NM_133919	*Aff1*	+	*
BE956940	*Lonrf3*	++	**	NM_011627	*Tpbg*	+	*	NM_144549	*Trib1*	++	*
BG069873	*Gnb1l*	−	*	NM_011817	*Gadd45γ*	+++	*	NM_144907	*Sesn2*	+	*
BG071037	*BC049807*	+	**	NM_011982	*Homer1*	+++	**	NM_145066	*Gpr85*	+	**
BG917504	*Btaf1*	+	*	NM_011997	*Casp8ap2*	−	*	NM_145150	*Prc1*	+	*
BM242340	*Pam*	+	**	NM_013498	*Crem*	+	*	NM_145463	*Tmem46*	++	*
BM939903	*Fbxo33*	++	*	NM_013504	*Dsc1*	+	*	NM_145839	*Rasgef1b*	++	*
BM941356	*Pcdh9*	++	**	NM_013562	*Ifrd1*	+	*	NM_145950	*Osgin2*	+	*
BQ032894	*Thrap1*	+	**	NM_013598	*Kitl*	+	*	NM_153155	*C1ql3*	+	**
BQ175781		−	*	NM_013613	*Nr4a2*	++	*	NM_153287	*Axud1*	++	*
BQ176303	*9630037P07Rik*	+	*	NM_013628	*Pcsk1*	+	*	NM_153401	*Tieg3*	−	*
BQ176417	*Cdh9*	+	*	NM_013642	*Dusp1*	++	*	NM_153553	*Npas4*	+++	***
M13227	*Penk1*	++	**	NM_013862	*Rabgap1l*	+	**	NM_172576	*Baz1a*	++	**
NM_007498	*Atf3*	+++	**	NM_015774	*Ero1l*	+	*	NM_175191	*Gpr22*	+	*
NM_007540	*Bdnf*	+	*	NM_015828	*Gne*	+	*	NM_175312	*B630005N14Rik*	+	*
NM_007553	*Bmp2*	+	**	NM_016789	*Nptx2*	+	**	NM_175341	*Mbnl2*	+	*
NM_007570	*Btg2*	+++	Ref.	NM_016870	*Exod1*	−	*	NM_175428	*Zfp295*	+	*
NM_007679	*Cebpd*	+	*	NM_017373	*Nfil3*	++	*	NM_175502	*Tmem74*	+	*
NM_007681	*Cenpa*	+	*	NM_018790	*Arc*	+++	**	NM_175513	*Zfp804a*	+	*
NM_008005	*Fgf18*	−	*	NM_018820	*Sertad1*	++	*	NM_177075	*C030019I05Rik*	++	**
NM_008013	*Fgl2*	++	***	NM_018869	*Gprk5*	+	*	NM_177468	*Spty2d1*	+	*
NM_008017	*Smc2l1*	−	*	NM_019518	*Grasp*	+	*	NM_178892	*Tiparp*	++	**
NM_008036	*Fosb*	+++	*	NM_019927	*Arih1*	+	*	NM_181750	*R3hdm1*	+	*
NM_008157	*Gpr19*	+	*	NM_019960	*Hspb3*	+	*	NM_183029	*Igf2bp2*	+	*
NM_008327	*Ifi202b*	+++	***	NM_019978	*Dcamkl1*	+	*	NM_199022	*Shc4*	+	*
NM_008343	*Igfbp3*	+	*	NM_019986	*Habp4*	+	*	NM_201531	*Kcnf1*	+	*
NM_008380	*Inhba*	+++	***	NM_020265	*Dkk2*	+	**				

Underlined genes have a putative role in cell death/cell survival based on information obtained using Gene Ontology (GO) analysis with the GO term ‘Apoptosis’ or literature searches. *AID* genes analyzed in this study are boxed. ID, GenBank ID. ACT/REP, activation/repression by action potential bursting. The fold changes compared to control in probe set expression are indicated as follows: significantly induced with fold changes between 2 and 5 (+), between 5 and 10 (++), and more than 10 (+++); significantly reduced with fold changes between 2 and 5 (−), between 5 and 10 (−−), and more than 10 (−−−). INH, percentage of inhibition of activation/repression by blockade of nuclear calcium signaling with CaMBP4 as indicated as follows: inhibition between 40 and 60% (*), between 60 and 80% (**), and more than 80% (***). Time point of analysis was 4 hours after induction of action potential bursting using bicuculline except for *Btg2* for which the time point for the fold induction given was 2 hours after induction of action potential bursting using bicuculline [6]. Ref. indicates that the data presented were from [6].

### Nuclear calcium–regulated survival genes

Because nuclear calcium signals evoked by AP bursting strongly promote neuronal survival [2]–[4],[6], we next aimed at identifying putative neuroprotective genes present in the nuclear calcium-regulated gene pool. Using a Gene Ontology (GO) analysis with the GO term ‘Apoptosis’ and a literature search we were able to identify 20 nuclear calcium-regulated genes that have been implicated in cell death/survival processes in non-neuronal or neuronal cells (Table 1). Our attention was drawn in particular to 8 nuclear calcium-regulated genes that, based on the microarray data, showed a very robust induction (more than 10 fold changes) of expression following neuronal activity. This includes 6 genes with known or putative functions in the cell nucleus (*Atf3*, *GADD45β*, *GADD45γ*, *Interferon activated gene 202b* (*Ifi202b*), *Npas4*, and *Nr4a1*) and 2 genes encoding secreted proteins (*Inhibin β-A* and *Serpinb2*). *Atf3* (activating transcription factor 3) is a member of the ATF/cAMP-responsive element-binding protein (CREB) family of transcription factors [15] that has been implicated in survival processes in neuronal and non-neuronal cells [16]–[18]. The family of growth arrest and DNA damage-inducible 45 (*GADD45*) genes comprises three members (*GADD45α*, *GADD45β* and *GADD45γ*) that are expressed in response to stress stimuli and DNA damage. *GADD45* genes have been implicated in DNA excision-repair processes [19],[20] but may also contribute to gene transcription via a process that involves DNA demethylation [21]. *Ifi202b* belongs to the interferon-activated p202 gene family that plays a role in cell survival and the regulation of caspase activation [22],[23]. *Npas4* (also known as *NxF*) is a member of the basic helix-loop-helix/Per-Arnt-Sim (bHLH-PAS) homology protein family [24]; it functions as a transcriptional regulator with possible roles in cell survival and differentiation [25],[26]. *Nr4a1* (also known as *nur77* or *NGFIB*) is an orphan nuclear hormone receptor with possible pro-apoptotic and anti-apoptotic functions [27]–[29]. *Serpinb2* [also known as plasminogen activator inhibitor type-2 (*PAI-2*)] is a serine proteinase inhibitor that can influence cell proliferation, differentiation and cell death [30],[31]. *Inhibin β-A* is a member of the transforming growth factor (TGF)-β superfamily [32] that can protect human SH-SY5Y neuroblastoma cells from chemical-induced death and may mediate neuroprotective actions of basic FGF [33],[34]. We considered these 8 genes plus the previously identified pro-survival gene *Btg2* (which is robustly induced by neuronal activity in a nuclear calcium-dependent manner [6]) as the core components of the putative neuroprotective gene program and refer to this set of genes hereafter as *Activity-regulated Inhibitor of Death (AID)* genes (Table 1; *AID* genes are boxed).

**Figure 2:**
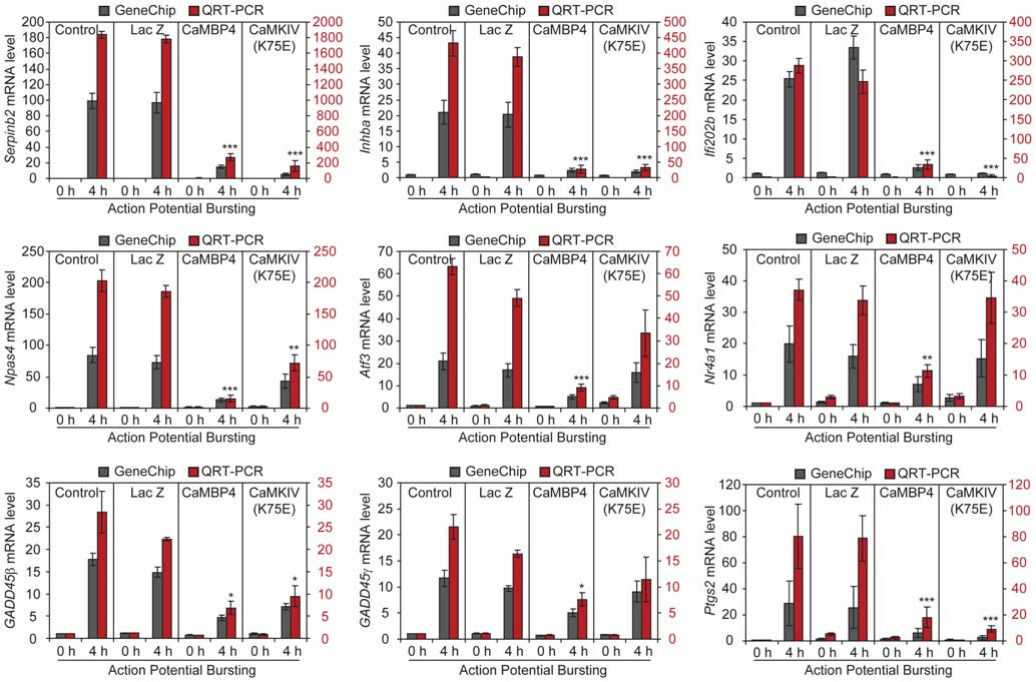
Nuclear calcium signaling controls activity-dependent regulation of *AID* genes. Affymetrix GeneChip profiles and QRT-PCR analysis of the indicated *AID* genes and *Ptgs2* are shown. Uninfected hippocampal neurons or hippocampal neurons infected with rAAV-*LacZ*, rAAV-*CaMBP4*, or rAAV-*CaMKIVK75E* were left unstimulated or were stimulated for 4 hours with 50 µM bicuculline (to induce AP bursting). Total RNA extracted from hippocampal neurons was used for GeneChip expression profiling and QRT–PCR analysis. Bars represent means±SEM (GeneChip, *n* = 3; QRT–PCR, *n* = 4). Statistical analysis of the inhibition of the AP bursting-induced increase in mRNA expression, assessed using QRT-PCR, by CaMBP4 or CaMKIVK75E relative to rAAV-*LacZ* control was determined by analysis of variance (ANOVA); *p<0.05; **p<0.01; ***p<0.001. The significance of the AP bursting-induced increase in expression (compared to unstimulated control), assessed using QRT–PCR, in uninfected hippocampal neurons and in hippocampal neurons infected with rAAV-*LacZ* was p<0.001 for each gene shown.

Using QRT-PCR, we confirmed the regulation of each *AID* gene by AP bursting and nuclear calcium signaling; the regulation of *Btg2* by nuclear calcium signaling has been established previously [6]. The expression of *GADD45β*, *GADD4γ*, and *Nr4a1* increased about 20 to 35 fold after AP bursting, which were the weakest inductions among this group of genes (Figure 2). Significantly higher fold changes relative to unstimulated control in either uninfected or rAAV-*LacZ* infected hippocampal neurons were observed for *Atf3* (63±4 fold, uninfected; 49±4 fold, rAAV-*LacZ*), *Npas4* (203±17 fold, uninfected; 186±9 fold, rAAV-*LacZ*), *Ifi202b* (288±19 fold, uninfected; 246±30 fold, rAAV-*LacZ*), and *Inhibin β-A* (432±41 fold, uninfected; 388±30 fold, rAAV-*LacZ*) (Figure 2). For *Serpinb2*, QRT-PCR revealed a very dramatic, 1839±45 fold increase in expression following AP bursting in uninfected hippocampal neurons and 1781±48 fold increase in rAAV-*LacZ* infected hippocampal neurons (Figure 2). This is - to the best of our knowledge - the highest fold change ever observed for a signal–regulated gene. For all *AID* genes, we confirmed the requirement for nuclear calcium signaling for their induction by AP bursting (Figure 2). Most dramatic inhibitions of well over 80 percent were observed for *Inhibin β-A* (93±3% inhibition), *Npas4* (92±3% inhibition), *Ifi202b* (87±4% inhibition), and *Serpinb2* (85±3% inhibition), and *Atf3* (80±5%) (Figure 2). We also included prostaglandin-endoperoxide synthase 2 (*Ptgs2*; also known as *Cox2*) in the QRT-PCR analysis. Expression of *Ptgs2* is robustly induced by neuronal activity (80±24 fold, uninfected; 78±18 fold, rAAV-*LacZ*), and this induction was inhibited by CaMBP4 by 68±20% (Figure 2). Because there is no available evidence for a role of *Ptgs2* in promoting survival of neuronal or non-neuronal cells, this gene served as one of the negative controls in the *in vivo* survival experiments (see below).

Given the importance of CREB and its co-activator CREB binding protein (CBP) in mediating transcriptional activation by synaptic activity and nuclear calcium signaling [12],[35],[36] and the critical role of CREB in neuronal survival [3],[37],[38], we carried out data base searches to determine whether the nuclear calcium-regulated genes, in particular *AID* genes, contain putative CREB binding sites. Information retrieved from two databases (the CREB ‘regulon’ (http://saco.ohsu.edu/) [39] and the CREB target gene database (http://natural.salk.edu/creb/) [40] indicated that a large fraction of the nuclear calcium regulated gene pool (56%) and all *AID* genes except *Npas4* contain one or several CREs or CRE-like sequences, suggesting that they could be CREB target genes (Table S1). In addition to CREB, other CBP-recruiting transcription factors may contribute to the regulation of *AID* genes by nuclear calcium signaling.

**Figure 3:**
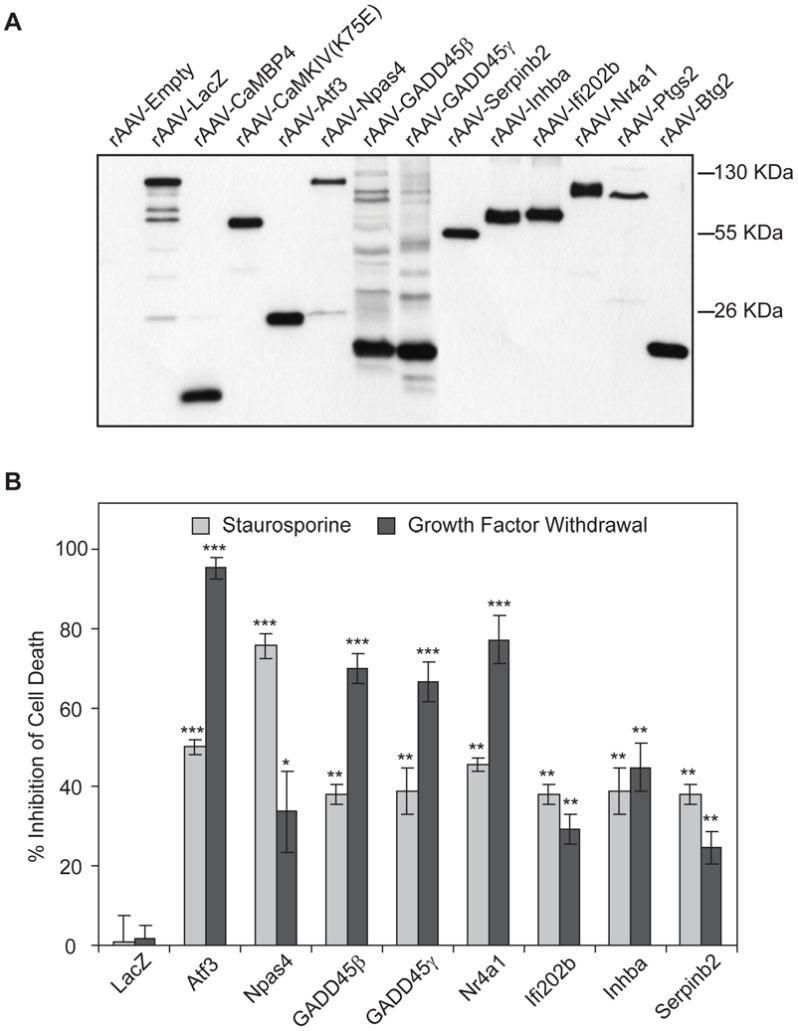
*AID* genes promote neuronal survival *in vitro*. (A) Immunoblot analysis of viral vector mediated genes expression in hippocampal neurons. Hippocampal neurons were infected with rAAV-*Empty*, rAAV-*LacZ*, rAAV-*CaMBP4*, rAAV-*CaMKIVK75E*, rAAV-*Npas4*, rAAV-*Nr4a1*, rAAV-*Atf3*, rAAV-*Ifi202b*, rAAV-*GADD45β*, rAAV-*GADD45γ*, rAAV-*Inhba*, rAAV-*Serpinb2*, rAAV-*Ptgs2*, or rAAV-*Btg2* and Flag-tagged proteins were detected with anti-Flag antibodies. (B) Analysis of cell death induced by growth factor withdrawal or staurosporine treatment in hippocampal neurons infected with rAAVs expressing Atf3, GADD45β, GADD45γ, Ifi202b, Inhibin β-A, LacZ, Npas4, Nr4a1, and Serpinb2. The growth factor withdrawal- and staurosporine-induced increase in dead cells in infected neurons relative to the growth factor withdrawal- and staurosporine-induced increase in dead cells in uninfected neurons was calculated; the effects of viral vector-mediated expression of the indicated genes is shown as percentage inhibition of cell death relative to uninfected control. Bars represent means±SEM (*n* = 3). Statistical analysis was determined by analysis of variance (ANOVA); **p<0.01; ***p<0.001.

We next investigated the role of the nuclear calcium/calmodulin-dependent protein kinase IV (CaMKIV) in the regulation of *AID* genes. CaMKIV is one important mediator of nuclear calcium/CREB-regulated transcription [36], [41]–[46]. To inhibit CaMKIV activity, we infected hippocampal neurons with a rAAV containing an expression cassette for a kinase-inactive form of CaMKIV (*CaMKIVK75E*) that functions as a negative interfering mutant of CaMKIV [36],[42],[43]; immunoblot analysis of expression of *CaMKIVK75E* in hippocampal neurons infected with rAAV-CaMKIVK75E is shown in Figure 3A. We found that in hippocampal neurons infected with rAAV-CaMKIVK75E the induction by AP bursting of all *AID* genes and induction of *Ptgs2* was inhibited (Figure 2). For 5 *AID* genes (i.e. *GADD45β*, *GADD45γ*, *Serpinb2*, *Inhibin β-A*, and *Ifi202b*) and for *Ptgs2*, the percent inhibition by rAAV-*CaMKIVK75E* was very similar to the inhibition obtained by the blockade of nuclear calcium signaling using CaMBP4 (Figure 2). CaMKIVK75E was slightly less potent than CaMBP4 in inhibiting induction of *Atf3*, *Npas4*, and *Nr4a1* (Figure 2), suggesting that targets of nuclear calcium other than CaMKIV may contribute to regulation of these genes by neuronal activity. These results indicate that nuclear calcium-CaMKIV is an important regulatory module of *AID* genes.

### *AID* genes promote neuronal survival *in vitro*

We next investigated the role of *AID* genes in neuronal survival. We first carried out gain-of-function experiments in which we used rAAV-mediated gene delivery to over-express Flag-tagged *AID* proteins in cultured hippocampal neurons. Expression of the proteins was assessed with immunoblots using antibodies to the Flag-tag (Figure 3A). Infection rates were determined immunocytochemically and ranged from 80 to 95 percent of the viable neurons (data not shown). We used two types of assays to assess apoptotic cell death: growth factor withdrawal and treatment of cultured hippocampal neurons with a low concentration of staurosporine [2],[6], a classical inducer of apoptotic cell death. We found that compared to control (i.e. non-infected neurons or neurons infected with rAAV-*LacZ*), cell death induced by either growth factor withdrawal or staurosporine treatment was inhibited in neurons infected with rAAV carrying Flag-tagged *AID* proteins (Figure 3B). Inhibition of apoptosis ranged from about 30 to 95% for growth factor withdrawal-induced apoptosis and from about 40 to 80% for apoptosis induced by staurosporine (Figure 3B). Over-expression of Atf3, Nr4a1, GADD45β, and GADD45γ yielded the most potent inhibition for growth factor withdrawal-induced apoptosis, whereas expression of Npas4 was most efficient in protecting against staurosporine-induced apoptosis (Figure 3B). These results indicate that *AID* proteins can confer robust neuroprotection to cultured hippocampal neurons.

**Figure 4:**
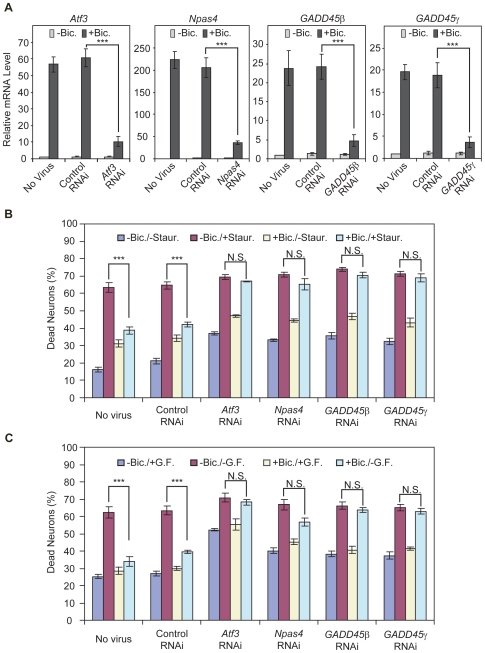
*AID* genes are necessary for activity-dependent neuronal survival. (A) QRT–PCR analyses illustrating the blockade of activity-dependent induction of *Npas4*, *Atf3*, *GADD45β* and *GADD45γ* using RNAi. Uninfected hippocampal neurons or hippocampal neurons infected with rAAVs expressing either a control shRNA (rAAV-Control-RNAi) or shRNAs that target *Atf3* (rAAV-*Atf3*-RNAi), *GADD45β* (rAAV-*GADD45β*-RNAi), *GADD45γ* (rAAV-*GADD45γ*-RNAi), or *Npas4* (rAAV-*Npas4*-RNAi) were stimulated for 4 hours with 50 µM bicuculline to induce AP bursting or were left unstimulated. Bars represent means±SEM (*n* = 3). Statistical analysis was determined by analysis of variance (ANOVA); ***p<0.001. For each gene shown, the significance for the AP bursting-induced increase in expression (compared to unstimulated control), in uninfected hippocampal neurons and in hippocampal neurons infected with rAAV-Control-RNAi was p<0.001 (ANOVA). (B,C) Analysis of the role of *Atf3*, *GADD45β*, *GADD45γ* and *Npas4* in activity-dependent neuronal survival. Uninfected hippocampal neurons or hippocampal neurons infected with rAAV-Control-RNAi, rAAV-*Atf3*-RNAi, rAAV-*GADD4β*-RNAi, rAAV-*GADD45γ*-RNAi and rAAV-*Npas4*-RNAi were left untreated or were treated for 16 hours with bicuculline (50 µM) in the presence of 4-AP (250 µM) to induce activity-dependent neuronal survival. Subsequently, apoptosis induced by staurosporine treatment (B) or growth factor withdrawal (-GF) (C) was analyzed. Expression of rAAV-*Atf3*-RNAi, rAAV-*GADD45β*-RNAi, rAAV-*GADD45γ*-RNAi or rAAV-*Npas4*-RNAi but not rAAV-Control-RNAi reduced neuroprotection afforded by synaptic activity. Bars represent means±SEM (B, *n* = 3; C, *n* = 3). Statistical analysis was determined by analysis of variance (ANOVA); ***p<0.001; N.S, not significant. The infection rates ranged from 85 to 95 percent of the neurons; they were determined immunocytochemically using antibodies to hrGFP or by analyzing the fluorescence of hrGFP. In both types of cell death assays, the basal cell death rates in hippocampal neurons expressing shRNAs specific for *Atf3*, *Npas4*, *GADD4β*, or *GADD45γ* were higher than the basal death rates obtained in hippocampal neurons expressing the control shRNA; for both cell death assays, p<0.01 for *Npas4*, *GADD45β*, or *GADD45γ* and p<0.001 for *Atf3*.

We next investigated whether *AID* genes contribute to activity-dependent survival induced by AP bursting and activation of synaptic NMDA receptors [2]–[4],[6]. For this analysis, we selected three genes (*Atf3*, *GADD45β*, and *GADD45γ*) that protected efficiently against growth factor withdrawal-induced apoptosis and *Npas4* that protected efficiently against staurosporine induced apoptosis (see Figure 3B). RNA interference (RNAi) was used to inhibit expression of these genes following synaptic activity. DNA sequences encoding short hairpin RNAs (shRNAs) designed to appropriate target regions were inserted downstream of the U6 promoter of a rAAV vector that also harbors an expression cassette for humanized *Renilla reniformis* green fluorescent protein (hrGFP) [6] (for details see Text S1). To control for non-specific effects of infections with rAAVs carrying an expression cassette for shRNAs, a rAAV was used that contains a universal control shRNA (rAAV-Control-RNAi), which has no significant sequence similarity to the mouse, rat, or human genome. For all rAAVs carrying an expression cassette for shRNAs, infection rates of 80 to 95 percent of the neuron population were obtained (data not shown). QRT-PCR analysis revealed that RNAi was effective in eliminating induction of *Atf3*, *GADD45β*, and *GADD45γ*, and *Npas4* by AP bursting in hippocampal neurons. The inhibition of expression was in the range of 85% for all 4 genes; rAAV-Control-RNAi had no significant effect (Figure 4A). Given the neurotropism of rAAVs used in the study [47], the results also indicate that the induction of these genes occurs in hippocampal neurons and not in glial cells.

To assess activity-dependent survival, apoptotic cells were counted after treatment with staurosporine or withdrawal of growth factors with and without previous periods of neuronal activity (Figure 4B and 4C) [2],[3],[6]. In the staurosporine assays, we detected about 10 to 15% of apoptotic cells in the control condition, which increased to about 60% after treatment (Figure 4B). In the growth factor withdrawal assays, basal cell death was slightly higher (about 20 to 25%) and also increased to about 60% (Figure 4C). Upon subjecting the neurons to a period of 12–16 hours of synaptic activity (induced by bicuculline treatment in the presence of 4-amino pyridine, which increases the burst frequency [2]) prior to growth factor withdrawal or staurosporine exposure, far fewer cells underwent apoptosis (Figure 4B and 4C). As shown previously, this activity-dependent survival is triggered by calcium entry into the neurons through synaptic NMDA receptors and involves nuclear calcium signaling [2]–[4],[6]. In uninfected neurons and neurons infected with rAAV-Control-RNAi, we observed the typical stimulus-induced increase in apoptotic cells; following the period of synaptic activity prior to staurosporine exposure or growth factor withdrawal, the stimulus-induced cell death was reduced (Figure 4B and 4C). In contrast, in neurons infected with rAAV-*Atf3*-RNAi, rAAV-*GADD45β*-RNAi, rAAV-*GADD45γ*-RNAi, and rAAV-*Npas4*-RNAi the basal level of cell death was slightly elevated and activity-dependent survival was severely compromised (Figure 4B and 4C). These results indicate that the *AID* genes *Atf3*, *GADD45β*, *GADD45γ* and *Npas4* are important for neuronal survival and represent key components of the synaptic NMDA receptor-induced genomic neuroprotective program.

### Neuroprotection through inhibition of death signal–induced mitochondrial permeability transition

**Figure 5:**
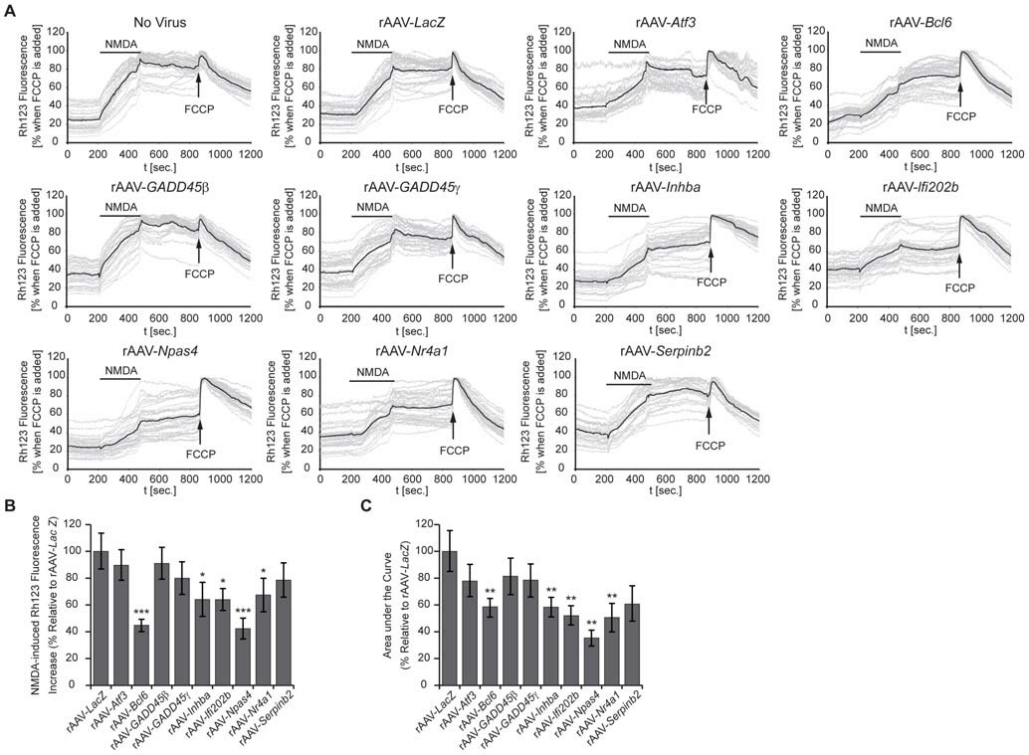
Rh123 imaging of NMDA–induced break-down of mitochondrial membrane potential. (A) Rh123 imaging of uninfected hippocampal neurons and hippocampal neurons infected with the indicated rAAVs. Neurons were stimulated with NMDA (30 µM) for 4 min followed by washout of NMDA and treatment at the indicated time with the mitochondrial uncoupler, FCCP (5 µM) to obtain the maximal Rh123 signals. Representative traces are shown; the thick line represents the mean value. (B,C) Quantitative analysis of Rh123 measurements. The NMDA-induced percent increase in Rh123 fluorescence after NMDA application is shown in (B). The area under the curve represents the integral of the Rh123 signals above baseline beginning at the time of NMDA application until the application of FCCP (C). Data represents mean±SEM (n≥4 independent experiments, with at least 100 single cells). Statistical analysis was determined by analysis of variance (ANOVA); *p<0.05; **p<0.01; ***p<0.001.

Virtually all cell death processes involve the deregulation of mitochondrial functions. One important early event in excitotoxic cell death is the collapse of the mitochondria membrane potential and the shift in the mitochondrial membrane permeability, known as mitochondrial permeability transition (MPT) [48]–[50]. Using Rhodamine 123 (Rh123) imaging techniques to monitor the mitochondrial membrane potential, we have recently shown that one mechanism through which down-regulation of the tumor suppressor gene, *p53*, or increasing expression of Btg2 can enhance the survival of hippocampal neurons involves inhibition of the NMDA-induced break-down of the mitochondrial membrane potentials [51]. We therefore investigated whether the identified *AID* genes can also act through a process that guards mitochondria against toxic insults. Rh123 imaging of uninfected hippocampal neurons and hippocampal neurons infected with rAAV-*LacZ* revealed the typical increase in Rh123 fluorescence after application of NMDA (30 µM), which is indicative of the break—down of mitochondrial membrane potential [2],[51],[52]. In contrast, in hippocampal neurons that had been infected with rAAV to express the *AID* genes *Npas4*, *Inhibin β-A*, *Ifi202b*, and *Nr4a1*, or to express the previously identified neuroprotective gene, *Bcl6* [6], the NMDA-induced loss of mitochondrial membrane potential occurred with slower kinetics and reached significantly lower magnitudes (Figure 5A–5C). A quantitative analysis of the imaging data revealed that expression of *Npas4* and *Bcl6* had the largest inhibitory effects on the NMDA-induced break—down of mitochondrial membrane potential (Figure 5B and 5C). The inhibitions by *Inhibin β-A*, *Ifi202b*, and *Nr4a1* were smaller but also significant, whereas no significant reductions were observed for *Atf3*, *GADD45β*, *GADD45γ*, and *Serpinb2* under the conditions used (Figure 5B and 5C). These results suggest that one converging point common to several *AID* genes is the mitochondria, which are rendered more resistant against death signal-induced dysfunction.

### *AID* genes protect against seizure-induced neuronal death

**Figure 6:**
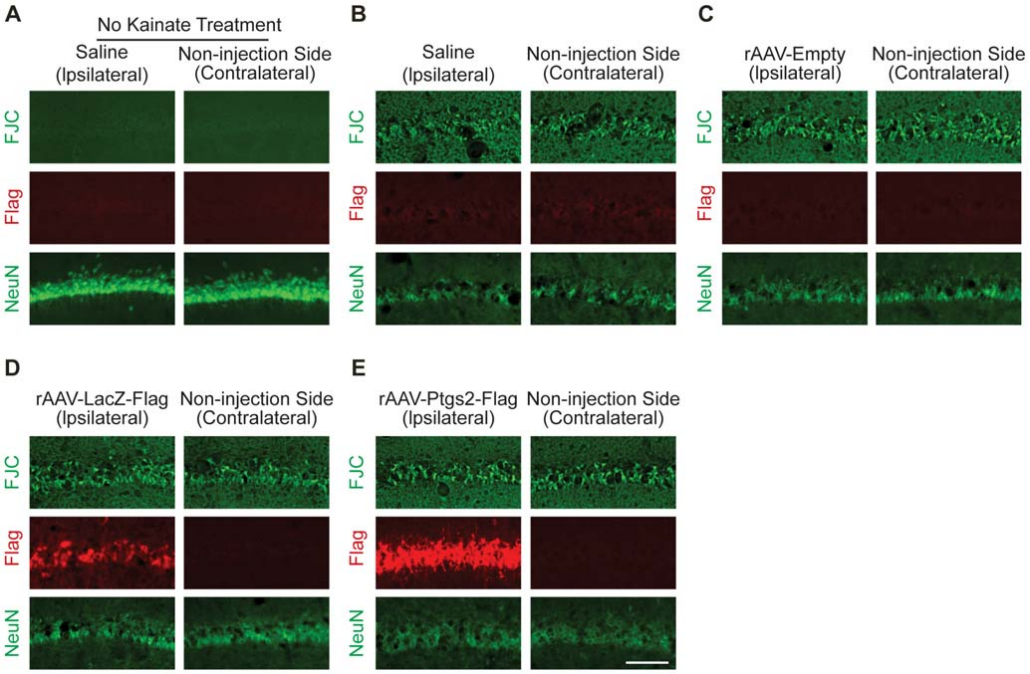
Kainate-induced neuronal cell death in control groups. Kainate-induced cell death was assessed by Fluoro-Jade C labeling and NeuN immunoreactivity in the CA1 region of the rat hippocampus. (A) Without systemic administration of kainate to rats that have been stereotaxically injected unilaterally with PBS into the dorsal hippocampus, there was no Fluoro-Jade C staining in CA1 of the hippocampus of the ipsilateral (i.e. injected) and the contralateral (i.e. the non-injected) hemispheres. (B–E) Robust Fluoro-Jade C staining in the CA1 pyramidal cell layer was detected three days after systemic administration of kainate to rats that have been stereotaxically injected unilaterally into the dorsal hippocampus with PBS (saline, B), rAAV-*Empty* (C), rAAV-*LacZ* (D), rAAV-*Ptgs2* (E) two weeks prior to kainate treatment. The ipsilateral (i.e. injected) hemispheres and the contralateral (i.e. the non-injected) hemispheres are shown. Viral vector-mediated expression of Flag-tagged Ptgs2 and LacZ was detected using antibodies to the Flag tag. Neurons were labeled using the neuronal marker NeuN. Note the reduction in NeuN staining three days after systemic administration of kainate indicating dramatic loss of neurons (compare NeuN immunoreactivity in (A) with that in (B) through (E)). Representative examples are shown from (A, n = 3; B, n = 9; C, n = 9; D, n = 12; E, n = 12). A quantitative analysis of the Fluoro-Jade C staining is given in Table 2. Scale bar is 100 µm.

**Figure 7:**
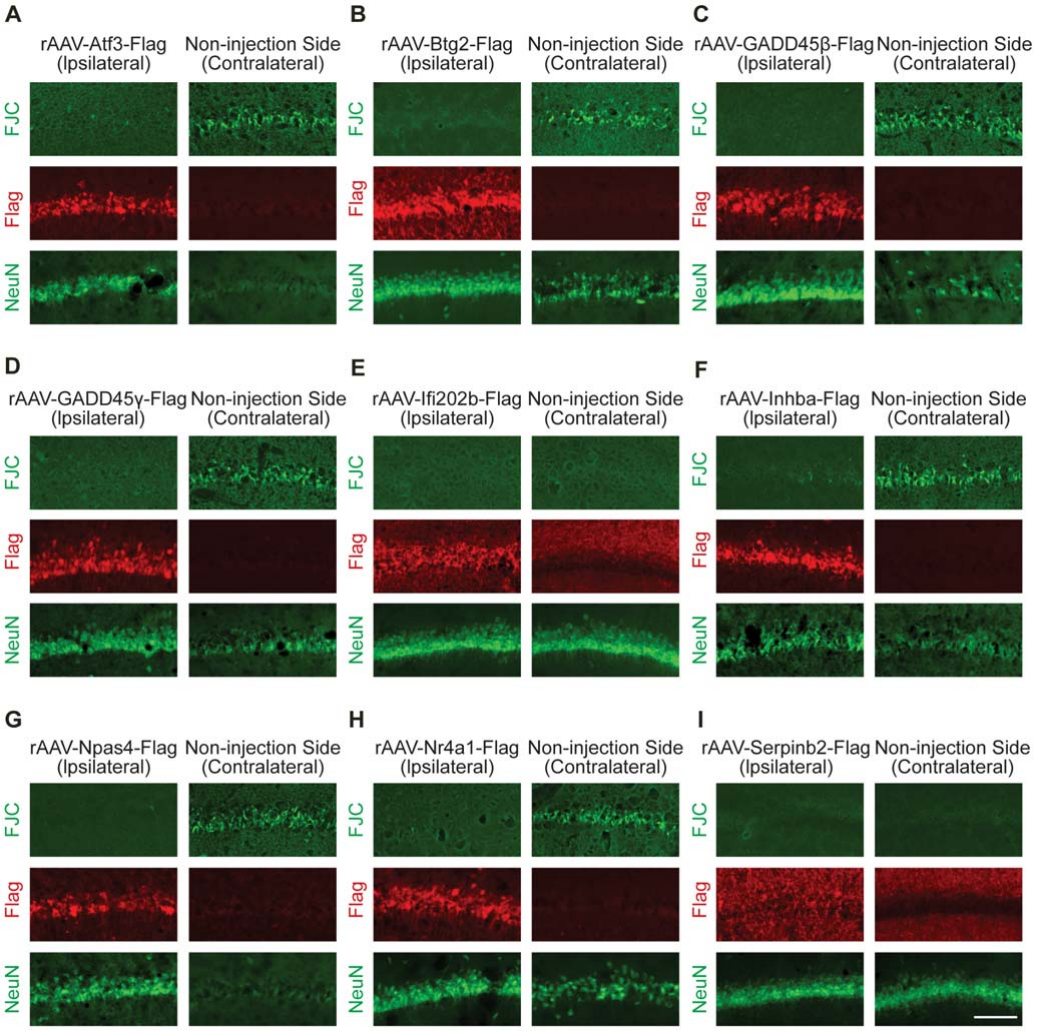
*AID* genes provide neuroprotection *in vivo*. Analysis of kainate-induced cell death in CA1 hippocampal neurons over-expressing Flag-tagged Atf3, Btg2, GADD45β, GADD45γ, Ifi202b, Inhibin β-A, Npas4, Nr4a1, and Serpinb2. Degeneration of neurons and cell loss in the CA1 region of the hippocampus was assessed using Fluoro-Jade C labeling and NeuN immunoreactivity three days after systemic administration of kainic acid to rats that have been stereotaxically injected unilaterally into the dorsal hippocampus with rAAV-*Atf3*, rAAV-*Btg2*, rAAV-*GADD45β*, rAAV-*GADD4γ*, rAAV-*Ifi202b*, rAAV-*Inhba*, rAAV-*Npas4*, rAAV-*Nr4a1*, or rAAV-*Serpinb2* two weeks prior to kainate treatment. The ipsilateral (i.e. injected) hemisphere and the contralateral (i.e. the non-injected) hemisphere are shown. Viral vector-mediated expression of Flag-tagged Atf3, Btg2, GADD45β, GADD45γ, Ifi202b, Inhibin-βA, Npas4, Nr4a1, and Serpinb2 was detected using antibodies to the Flag tag. Note the decrease of Fluoro-Jade C labeling and the stronger NeuN immunoreactivity on the ipsilateral (i.e. injected) hemisphere relative to the contralateral (i.e. the non-injected) hemisphere. In case of rAAV-*Ifi202b* and rAAV-*Serpinb2*, Ifi202b and Serpinb2 were consistently expressed also in the hippocampus of the contralateral (i.e. the non-injected) hemisphere and protected neurons from kainate-induced death. Representative examples are shown (n = 12). A quantitative analysis of the Fluoro-Jade C staining is given in Table 2. Scale bar is 100 µm.

We next analyzed the neuroprotective activity of *AID* genes *in vivo*. Stereotaxic injection was used to deliver rAAVs carrying expression cassettes for *AID* genes or appropriate negative controls (i.e. rAAV-*LacZ* and rAAV-*Empty*) to the dorsal hippocampus of male Sprague-Dawley rats weighing 230 to 250 g. We also included *Ptgs2* in our *in vivo* analysis as an additional negative control. Expression of *Ptgs2* is robustly induced by neuronal activity in a nuclear calcium/CaMKIV dependent manner (Figure 2). However, there is no available evidence for a role of *Ptgs2* in promoting survival of neuronal or non-neuronal cells and we therefore expected that expression of *Ptgs2 in vivo* would not provide neuroprotection. Two weeks after viral delivery, the rats were injected intra-peritoneally with kainic acid (KA), which induces seizures leading to cell death in the hippocampus [53]. The animals were sacrificed three days after KA injection. The brains were removed, cut into slices, and stained with the histofluorescent label, Fluoro-Jade C, which serves as a very reliable marker for degenerating neurons [54]. The slices were immunostained with antibodies to the neuronal marker, NeuN, and antibodies to the Flag tag to detect the over-expressed proteins. We found widespread KA-induced cell death in the CA1 region of the hippocampus of animals injected with rAAV-*Empty*, rAAV-*LacZ*, and rAAV-*Ptgs2*, as well as in the CA1 region of the non-injected side of the hippocampus (Figure 6). In contrast, expression of *AID* genes in the CA1 area of the injected side of the hippocampus protected against KA-induced cell death (Figure 7). Quantification of the Fluoro-Jade C signals revealed inhibitions of KA-induced cell death of 85±7% (*Atf3*), 87±5% (*Btg2*), 92±6% (*GADD45β*), 93±2 (*GADD45γ*), 96±1% (*Ifi202b*), 70±8% (*Inhibin β-A*), 92±3% (*Npas4*), 90±7% (*Nr4a1*), and 96±1% (*Serpinb2*); over-expression of *Ptgs2* or *LacZ* did not inhibit KA-induced cell death (Table 2). Expression of *Btg2*, *Ifi202b*, and *Serpinb2* consistently led to neuroprotection also on the contralateral (i.e. non-injected) side (Figure 7, Table 2). This could be due to secretion of the neuroprotective protein, which may be the case for the serine proteinase inhibitor, Serpinb2. It is also conceivable that expression of neuroprotective proteins in the processes of infected neurons, which project to the contralateral hippocampus, can promote survival of target neuron; this may be the case for flag-tagged Ifi202b which is readily detectable in the hippocampus of the non-injected, contralateral side (see Figure 7).

**Table 2:** Neuroprotective activities of *AID* genes *in vivo*.

Group	Stereotaxic	Kainate	Injected Side	Non-Injected Side
	Injection		FJC Signal (% Control)	FJC Signal (% Control)
***Control***	Saline	−	2.9±1.8	3.4±2.1
	Saline	+	100.1±10.9	96.5±15.6
	rAAV-Empty	+	126.0±28.3	124.8±22.8
	rAAV-*LacZ*	+	137.8±7.8	122.9±15.0
	rAAV-*Ptgs2*	+	127.3±19.4	131.3±6.4
***AID genes***	rAAV-*Atf3*	+	15.4±6.5 ***	86.4±7.2
	rAAV-*Btg2*	+	13.1±5.3 ***	44.1±11.6 **
	rAAV-*GADD45β*	+	8.1±6.2 ***	84.4±15.3
	rAAV-*GADD45γ*	+	7.5±2.1 ***	103.8±21.5
	rAAV-*Ifi202b*	+	4.4±0.7 ***	5.7±0.7 ***
	rAAV-*Inhba*	+	30.0±7.7 ***	83.5±8.3
	rAAV-*Npas4*	+	8.4±3.4 ***	97.0±21.5
	rAAV-*Nr4a1*	+	10.1±6.6 ***	113.3±25.5
	rAAV-*Serpinb2*	+	3.7±1.3 ***	4.5±0.5 ***

A quantitative analysis of the Fluoro-Jade C staining in the CA1 area of the hippocampus of non-injected and control-injected animals and animals stereotaxically injected with rAAVs expressing *AID* genes. The Fluoro-Jade C signal obtained without and with kainate treatment in the hippocampus of both the injected and the non-injected side is expressed as a percentage of control, i.e. the average kainate-induced Fluoro-Jade C signal obtained from the non-injected hemispheres of kainate treated animals (for details see Methods). The mean±SEM is given; compared to control condition ‘saline plus kainate’, each *AID* gene significantly reduced the Fluoro-Jade C signal (t-test; **p<0.01; ***p<0.001).

## Discussion

Neuronal activity boosts neuroprotection. Our study has identified the key players in this process. Using a strategy that combined the identification of all genes controlled by neuronal activity and nuclear calcium signaling with subsequent bioinformatics filtering procedures, a set of genes was unearthed that provides neurons with a robust neuroprotective shield. The core neuroprotective program contains 9 genes, which are referred to as *AID* genes.

### Nuclear calcium: key player in the dialogue between synapse and nucleus in neuronal survival

Activity-dependent, long-lasting neuroprotection as well as other adaptive responses in the nervous system require the dialogue between the synapse and the nucleus. There is growing evidence to suggest that calcium signals propagating from their site of activation at the plasma membrane towards the cell soma and the nucleus are key mediators of synapse-to-nucleus communication. Nuclear calcium, most likely acting via nuclear calcium/calmodulin dependent protein kinases, controls CREB/CBP-dependent transcription [12], [35], [36], [43]–[46] and is thought to regulate genomic programs critical for neuronal survival, synaptic plasticity, memory formation, and emotional behavior [3], [6], [55]–[59]. In this study, we identified the nuclear calcium-regulated gene pool in hippocampal neurons. The large number of 185 genes induced or repressed by nuclear calcium signaling is not unexpected. A genome-wide analysis revealed that CREB – the principal target of nuclear calcium signaling - can occupy between 4000 and 6000 promoter sites in the rat or human genome, although the transcription of only a subset of those genes is signal-induced in a given cell type perhaps due to preferential recruitment of CBP [39],[40],[60]. Indeed, 56% of nuclear calcium-regulated genes and all *AID* genes except *Npas4* are known or putative CREB targets (Table S1), underscoring the importance of the nuclear calcium-CREB axis in neuronal survival.

### Possible common targets and end points of neuroprotection

The *AID* genes characterized in this study fall into two functional categories: regulators of gene transcription and secreted proteins. Although they may act in concert to collectively provide full neuroprotection, over-expression of individual *AID* genes is sufficient to promote survival. Moreover, RNAi-based loss-of-function experiments indicate that the selective reduction of individual *AID* genes can compromise the activity-induced build-up of a neuroprotective shield. These results could be explained by a possible convergence of *AID* genes on one or a small number of targets that execute protection. Given that 7 out of 9 *AID* genes are putative regulators of gene expression, the existence of common target genes that are part of a ‘second wave’ transcriptional response is conceivable. Under physiological condition, a signal-regulated, coordinate induction of transiently expressed genes may be required for the regulation of common targets; thus interference with one or a small number of *AID* genes would disturb the system. However, high-level, constitutive expression of individual *AID* genes may be sufficient to activate or inactivate down-stream regulators of survival. The regulation of a putative common target could involve direct trans-activation by *AID* gene products through binding to the target genes' promoter elements, although other modes of regulation (such as control of mRNA or protein stability or activity-regulating post-translational modifications) are conceivable and may involve secondary responses triggered by *AID* genes. Additional components of such a regulatory network may include other survival-promoting transcriptional regulators such as *C/EBPβ* [61]–[64], or the secreted *AID* genes *Serpinb2* and *Inhibin β-A*, or *Bdnf* [65] (see Table 1); these genes may contribute through transcription-dependent and transcription-independent processes to the funneling of information flow and the reduction of complexity to few molecules implementing neuroprotection. Mitochondria, which are vital for supplying the energy required to maintain life, may be the end-point of neuroprotective processes. In this study we show that expression of *AID* genes renders the mitochondria of hippocampal neurons more resistant to harmful conditions. Thus, neuroprotection may ultimately guard mitochondria against stress and toxic insults to prevent mitochondrial dysfunction.

### Dysfunction of nuclear calcium–regulated survival: a unifying theme in neurodegenerative disorders and aging

The finding that activation of synaptic NMDA receptors and calcium signaling to the cell nucleus builds up a strong and lasting neuroprotective shield may change our view of neurodegenerative disorders and cell death associated with aging. Proper functioning of the endogenous neuroprotective machinery requires a sequence of events that can be disturbed at the level of synaptic transmission, synaptic NMDA receptor activation, the generation of calcium signal and their propagation to the cell nucleus, and the regulation of gene transcription. Malfunctioning of calcium signaling towards and within the cell nucleus may lead to neurodegeneration and neuronal cell death. In Alzheimer's disease cell death may be caused by compromised endogenous neuroprotection due to impaired synaptic transmission and synapse loss caused by A-*β* or changes in calcium homeostasis or calcium signaling in neurons expressing mutant presenilin-1 [66]–[71]. Consistent with this concept is the observation that compared to an age-matched healthy control group, individuals with Alzheimer's disease have reduced levels of the activated (i.e. phosphorylated) form of CREB [72]; calcium signaling to the cell nucleus is the key inducer of CREB phosphorylation on its activator site serine 133 [12]. Similarly, in aged neurons, calcium signaling may be altered at the level of calcium signal generation and/or calcium signal propagation [73]–[75]. This could explain the reduced levels of serine 133-phosphorylated CREB in the hippocampus of aged, learning-impaired rats [76]–[78], which could lead to compromised endogenous neuroprotection, progressive cell loss and cognitive decline. The development of strategies to boost the endogenous neuroprotective machinery may lead to effective therapies of neurodegenerative condition. Both in disease and aging, health and functionality of neurons may be preserved by expressing *AID* genes or by restoring or enhancing key signals, in particular nuclear calcium.

## Methods

### Hippocampal cultures and stimulations

Hippocampal neurons from newborn C57/Black mice were cultured in Neurobasal media (Invitrogen, Carlsbad, CA, USA) containing 1% rat serum, B27 (Invitrogen, Carlsbad, CA, USA), and penicillin and streptomycin (Sigma). The procedure used to isolate and culture hippocampal neurons has been described [79],[80]. The hippocampal cultures used for this study typically contained about 10 to 15% glial cells and therefore a fraction of the RNA isolated from the cultures was derived from glial cells. Stimulations were done after a culturing period of 9 to 12 days during which hippocampal neurons develop a rich network of processes, express functional NMDA-type and AMPA/Kainate-type glutamate receptors, and form synaptic contacts [12],[81]. Action potential bursting was induced by treatment with the GABA_A_ receptor antagonist bicuculline (Sigma) (50 µM) as described previously [2],[11],[12]. In the survival experiments, neurons were treated for 16 hours with bicuculline in the presence of 250 µM 4-amino pyridine (4-AP; Calbiochem) [2]. 4-AP increases the frequency of the bicuculline-induced action potentials bursts, thereby enhancing nuclear calcium, CREB-mediated transcription, and activity-induced neuroprotection [2],[11],[12].

### Whole-genome transcription profiling

DNA microarray analysis was done using Affymetrix GeneChip Mouse Genome 430 2.0 Arrays. See **Text S1** for details.

### Recombinant adeno-associated virus (rAAV) and viral infections

The vectors used to construct and package rAAVs have been described previously [6]. The rAAV cassette for mRNA expression contains a CMV/chicken β actin hybrid promoter. The following rAAVs were generated and confirmed by DNA sequencing: rAAV-*Atf3*, rAAV-*CaMKIVK75E*, rAAV-*GADD45β*, rAAV-*GADD45γ*, rAAV-*Ifi202b*, rAAV-*Inhba*, rAAV-*LacZ*, rAAV-*Npas4*, rAAV-*Nr4a1*, rAAV-*Ptgs2*, and rAAV-*Serpinb2*. rAAV-*Btg2* and rAAV-*CaMBP4* have been described [6]. All rAAV-expressed proteins except hrGFP carry a Flag tag. For shRNA expression, a rAAV vector was used that contains the U6 promoter for shRNA expression and a CMV/chicken β actin hybrid promoter driving hrGFP expression [6]. For details on the construction of rAAVs expressing shRNAs see **Text S1**. Hippocampal neurons were infected with rAAVs at 4 days *in vitro* (DIV). Infection efficiencies were routinely determined immunocytochemically at 9 DIV or 10 DIV using antibodies to the Flag tag or to hrGFP or by analyzing the fluorescence of hrGFP; they ranged from 80 to 95 percent of the viable neurons [6].

### Quantitative reverse transcriptase PCR (QRT–PCR)

To determine the mRNA expression levels of *Atf3*, *GADD45β*, *GADD45γ*, *Ifi202b*, *Inhba*, *Npas4*, *Nr4a1*, *Ptgs2*, *Serpinb2*, *Gusb*, and glyceraldehyde-3-phosphate dehydrogenase (*Gapdh*), QRT-PCR was performed using real-time TaqMan technology with a sequence detection system model 7300 Real Time PCR System (Applied Biosystems, Foster City, California, USA). For further details, see Text S1.

### Assessment of cell death in hippocampal cultures

As described previously [2],[3], two types of assays were used to investigate apoptotic cell death and the protection from cell death afforded by a period of action potential bursting. At 10 DIV, activity-dependent survival was induced by treatment of the neurons for 16 hours with bicuculline (50 µM) and 4-AP (250 µM). All electrical activity of the network was subsequently stopped using tetrodotoxin (TTX; TOCRIS Bioscience) (1 µM) followed by keeping the cells either in regular medium (containing growth and trophic factors) with or without staurosporine (Calbiochem) (10 nM), or in medium lacking growth and trophic factors, all in the presence of TTX (1 µM). The principal growth and trophic factors in the regular, serum-free hippocampal medium [termed transfection medium (TM) [76]] are insulin, transferrin, and selenium. Staurosporine-induced and growth factor withdrawal-induced apoptosis was assessed after 36 hours and 72 hours, respectively, by determining the percentage of hippocampal neurons with shrunken cell body and large round chromatin clumps characteristic of apoptotic death [2],[3]. In the growth factor withdrawal assays, basal cell death is slightly higher due to the differences in the time that the neurons are kept in serum free, TTX-containing media. At least 20 visual fields from each coverslip (corresponding to 1500–2000 cells per coverslip) were counted with Hoechst 33258 (Serva) and the percentage of dead cells was determined. TUNEL assays (Roche, Mannheim, Germany) were done according the instructions provided by the manufacturer and were used to validate the analysis of cell death using the Hoechst 33258 stain. Photomicrographs of examples of healthy and apoptotic hippocampal neurons stained with TUNEL and with Hoechst 33258 are shown in Figure S1. All cell death analyses were done without knowledge of the treatment history of the cultures. All results are given as means±SEM; statistical significance was determined by ANOVA.

### Imaging of mitochondrial membrane potential

Imaging of mitochondrial membrane potential was done using Rhodamine 123 (Rh123; Molecular Probes, Eugene, OR) as described [2],[51],[52]. Imaging and data analysis were performed without knowing the experimental conditions. Quantitative measurements are given as means±SEM from n≥4 experiments, with at least 100 cells analyzed each. Statistical significance was determined by ANOVA.

### Stereotaxic injections

rAAVs were delivered by stereotaxic injection into the dorsal hippocampus of male Sprague-Dawley rats weighing 230–250 g. Rats were randomly grouped and anaesthetized with ketamine. A total volume of 3 µl containing 3×10^8^ genomic virus particles were injected unilaterally over a period of 30 min at the following coordinates relative to Bregma: anteroposterior, −3.8 mm; mediolateral, 2.8 mm; dorsoventral, −2.8 to −3.8 mm from the skull surface. Procedures were done in accordance with German guidelines for the care and use of laboratory animals and to the respective European Community Council Directive 86/609/EEC.

### Kainate-induced status seizures

Two weeks after rAAV delivery, rats were injected with kainate (Sigma; 10 mg/kg i.p.) or vehicle (phosphate-buffered saline, PBS), and monitored for at least 4 hours to categorize the severity of epileptic seizures according to following criteria: level 1, immobility; level 2, forelimb and/or tail extension, rigid posture; level 3, repetitive movements, head bobbing; level 4, rearing and falling; level 5, continuous rearing and falling; level 6, severe tonic-clonic seizure [82]. Only animals that exhibited at least level 4 or 5 of epileptic seizure behavior were analyzed further. Three days after seizure induction, animals were deeply anesthetized with an overdose of Nembutal (300 mg/kg), pre-perfused transcardially with PBS, and perfused with 200 ml of neutral phosphate buffered 10% formalin (Sigma). Brains were removed and post-fixed overnight in the same fixative solution. For cryoprotection, brains were incubated in 30% sucrose in PBS for 2 days. Brains were rapidly frozen on dry ice. Frozen sections (40 µm thick) were collected in PBS. Three consecutive sections separated by a 240 µm distance were used for immunostaining and Fluoro-Jade C staining (Histo-Chem, Inc., Jefferson, Arkansas, USA), which selectively stains degenerating neuronal **c**ell bodies and pro**c**esses, regardless of the me**c**hanism of **c**ell death. Transgene expression was detected with anti-Flag antibodies (1∶2500, M2 mouse monoclonal; Sigma). Neuronal cell loss was assessed with NeuN immunostaining (1∶500, mouse monoclonal; Chemicon). Immunostaining was done using standard procedures. Fluoro-Jade C staining was done as described previously [54]. Images of Fluoro-Jade C staining were taken and the signals in the CA1 area of the hippocampus were quantified. The quantification was performed without knowing the experimental conditions. Images (10× objective, 1600*1200 pixels) of Fluoro-Jade C stained sections were taken at central CA1 region infected with rAAVs. Sections were collected every 240 µm by cryostat sectioning at the level of 3.0∼5.0 mm posterior to Bregma. Five sections from each brain hemisphere were chosen for image analysis. Fluoro-Jade C signals from the images were quantified with NIH ImageJ software (National Institute of Health, Bethesda, MD, USA). Background intensity was measured from the CA1 area lacking positively-stained neuronal cell bodies. Threshold level was set as means of the background +3 SD. The CA1 pyramidal cell layer was encircled manually and particle analysis was performed. Particles were defined as 30 pixels to infinity, roundness 0∼1.0. Total pixel of Fluoro-Jade C positive particles from each section was obtained to calculate the mean cell death area for each hemisphere. All pixel values were normalized to the average kainate-induced Fluoro-Jade C signal from the control (i.e. non-injected) hemispheres of kainic acid treated animals; the signal obtained from the non-injected hemispheres of the animals injected with rAAV-*Btg2*, rAAV-*Ifi202b*, and rAAV-*Serpinb2* was not included into the calculation of the average kainate-induced Fluoro-Jade C signal because for rAAV-*Btg2*, rAAV-*Ifi202b*, and rAAV-*Serpinb2* we observed neuroprotection on the contralateral, non-injected hemispheres.

### Animals

All animal experiments were done in accordance with the international ethical guidelines for the care and use of laboratory animals and were approved by the local animal care committee of the Regierungspräsidium Karlsruhe. We minimized the number of animals used and their suffering.

## Supporting Information

Figure S1 Detection of dead hippocampal neurons using Hoechst 33258 staining and TUNEL staining. TUNEL-positive neurons were detected with the In Situ Cell Death Detection Kit (TMR red) as described by the manufacturer (Roche, Mannheim, Germany). Fluorescence images were acquired using a Leica SP2 confocal microscope. (A) Photomicrographs (low magnification) of confocal microscopy images of hippocampal neurons stained with Hoechst 33258 or with TUNEL stained. Neurons were exposed for 24 hours to 20 nM of staurosporine (‘Death-Inducing-Stimulus’) or were left untreated (‘No Treatment’). Scale bar is 20 µm. (B,C) Photomicrographs (high magnification) of confocal microscopy images of individual hippocampal neurons stained with Hoechst 33258 and with TUNEL. Representative examples of healthy, TUNEL-negative (B) and apoptotic, TUNEL-positive (C) neurons are shown. The pictures in (C) illustrate the broad spectrum of the characteristic morphological changes associated with apoptosis, which are readily identifiable using Hoechst 33258 staining. Scale bars, 10 µm.
(4.01 MB TIF)

Table S1 Nuclear calcium-regulated genes as putative CREB targets. * see Zhang et al., Proc Natl Acad Sci U S A 102: 4459–4464 and http://natural.salk.edu/creb/. * * see Impey et al., Cell 119: 1041–1054 and http://saco.ohsu.edu/.
(0.44 MB PDF)

Text S1 Supplemental Methods.
(0.05 MB DOC)
