# Supplementary figures and images for: Nuclear Calcium Signaling Controls Expression of a Large Gene Pool: Identification of a Gene Program for Acquired Neuroprotection Induced by Synaptic Activity
Source: PLoS Genet. 2009 Aug 14;5(8):e1000604. doi: 10.1371/journal.pgen.1000604 (PMC2718706; doi:10.1371/journal.pgen.1000604)

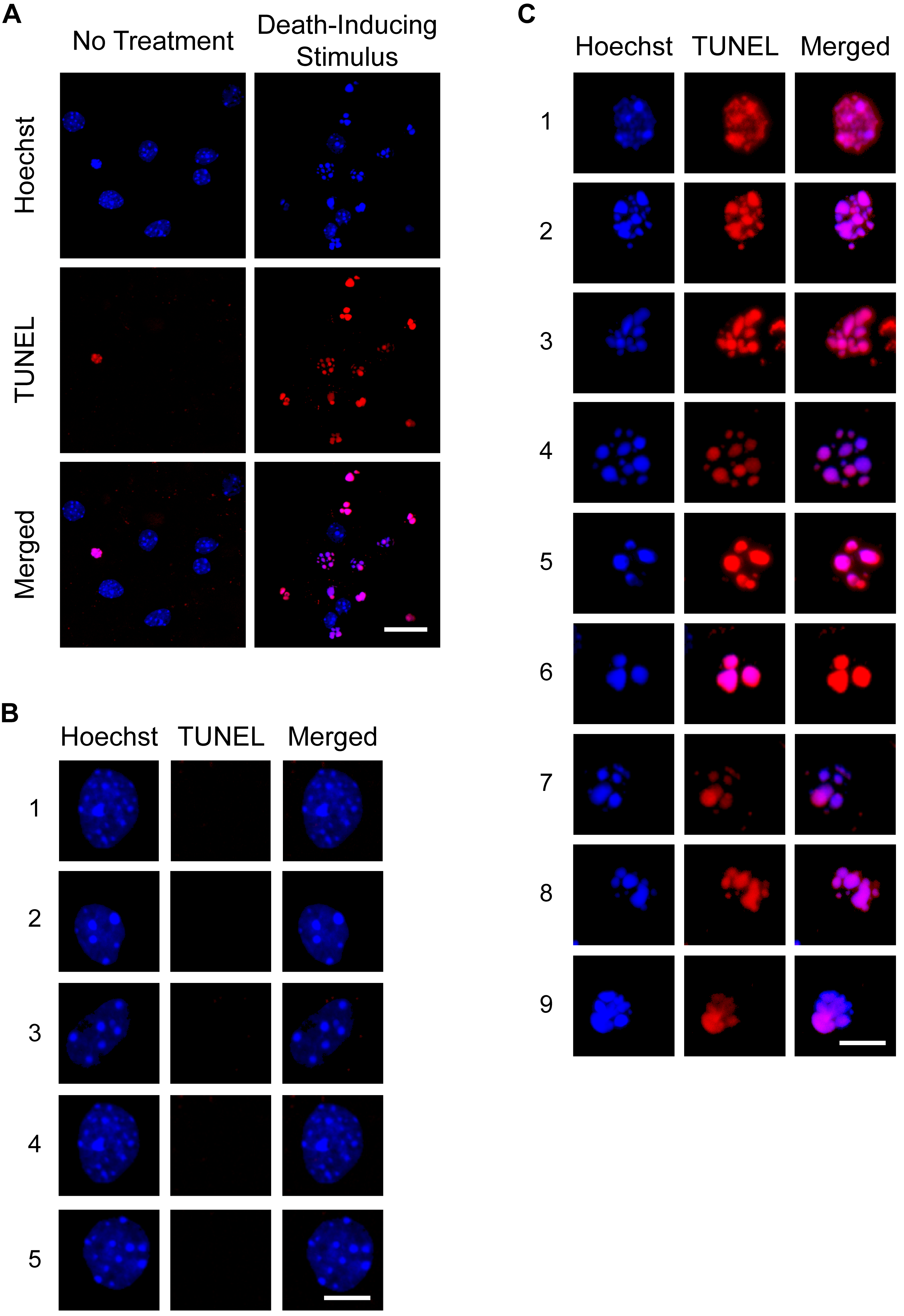

Supplement: Figure S1 — Detection of dead hippocampal neurons using Hoechst 33258 staining and TUNEL staining. TUNEL-positive neurons were detected with the In Situ Cell Death Detection Kit (TMR red) as described by the manufacturer (Roche, Mannheim, Germany). Fluorescence images were acquired using a Leica SP2 confocal microscope. (A) Photomicrographs (low magnification) of confocal microscopy images of hippocampal neurons stained with Hoechst 33258 or with TUNEL stained. Neurons were exposed for 24 hours to 20 nM of staurosporine (‘Death-Inducing-Stimulus’) or were left untreated (‘No Treatment’). Scale bar is 20 µm. (B,C) Photomicrographs (high magnification) of confocal microscopy images of individual hippocampal neurons stained with Hoechst 33258 and with TUNEL. Representative examples of healthy, TUNEL-negative (B) and apoptotic, TUNEL-positive (C) neurons are shown. The pictures in (C) illustrate the broad spectrum of the characteristic morphological changes associated with apoptosis, which are readily identifiable using Hoechst 33258 staining. Scale bars, 10 µm. (4.01 MB TIF) [file pgen.1000604.s001.tif]
