# Supplementary material for: Nuclear Calcium Signaling Controls Expression of a Large Gene Pool: Identification of a Gene Program for Acquired Neuroprotection Induced by Synaptic Activity
Source: PLoS Genet. 2009 Aug 14;5(8):e1000604. doi: 10.1371/journal.pgen.1000604 (PMC2718706; doi:10.1371/journal.pgen.1000604)
Supplement: Table S1 — Nuclear calcium-regulated genes as putative CREB targets. * see Zhang et al., Proc Natl Acad Sci U S A 102: 4459–4464 and http://natural.salk.edu/creb/. * * see Impey et al., Cell 119: 1041–1054 and http://saco.ohsu.edu/. (0.44 MB PDF) [file pgen.1000604.s002.pdf]

GenBank ID	Gene Name	Putative CREB Target*	CREB Target by ChIP**	GenBank ID	Gene Name	Putative CREB Target*	CREB Target by ChIP**	GenBank ID	Gene Name	Putative CREB Target*	CREB Target by ChIP**
AB019028	*Crsp2*	+		NM_008416	*Junb*	+	+	NM_021382	*Tacr3*	+	
AI507491	*1110020A10Rik*			NM_008543	*Smad7*	+		NM_021458	*Fzd3*	+	
AI597036				NM_008548	*Man1a*	+		NM_021462	*Mknk2*	+	
AK004371	*Rasl11a*			NM_008562	*Mcl1*	+		NM_021788	*Sap30*	+	
AK013051	*2810409C01Rik*			NM_008565	*Mcm4*	+		NM_022995	*Tmepai*		
AK020483	*Malat1*			NM_008655	*Gadd45β*	+		NM_023324	*Peli1*	+	
AK021003	*B230216N24Rik*			NM_008717	*Zfml*			NM_023503	*Ing2*	+	
AV309085	*1190002N15Rik*			NM_008780	*Pax1*	+		NM_024166	*2410018M08Rik*		
AV348660	*Gm1568*			NM_008842	*Pim1*	+	+	NM_024285	*Bves*	+	
AW109901	*4933411B09Rik*			NM_008872	*Plat*	+	+	NM_025404	*Arfl4*		
AW490446	*4930505D03Rik*			NM_008924	*Prkar2a*	+		NM_025413	*Lce1g*		
AW555393	*Mest*	+		NM_008965	*Ptger4*	+		NM_025635	*Zwint*	+	
BB009682	*4930434J08Rik*			NM_008987	*Ptx3*	+		NM_025775	*Tmtc2*		
BB013412	*6330417G02Rik*			NM_009013	*Rad51ap1*	+		NM_026153	*5730557B15Rik*	+	
BB096900	*4933402J24Rik*			NM_009044	*Rel*	+		NM_026324	*Kirrel3*	+	
BB161981				NM_009230	*Soat1*	+		NM_026394	*Lce1f*		
BB194610	*A330084C13Rik*			NM_009264	*Sprr1a*	+		NM_026989	*Sfrs11*		
BB246700	*A630072M18Rik*			NM_009551	*Zfand5*			NM_027514	*Pvr*	+	
BB253137	*Inhbb*			NM_009744	*Bcl6*	+		NM_027518	*6330416L11Rik*	+	
BB268139	*Ibrdc1*			NM_009769	*Klf5*	+	+	NM_027559	*BC063749*		
BB322180	*Phf21b*			NM_009859	*Sept7*			NM_028067	*Tsc22d2*		
BB322233	*5430433G21Rik*			NM_009883	*Cebpb*	+	+	NM_028755	*Arpp21*		
BB354702				NM_009911	*Cxcr4*	+		NM_028760	*Cep55*		
BB387595	*Gprin2*			NM_010193	*Fem1b*	+	+	NM_028829	*Paqr8*		
BB389395	*Onecut2*	+		NM_010207	*Fgfr2*	+		NM_029466	*Arl5b*		
BB398124	*C330006P03Rik*			NM_010276	*Gem*	+		NM_029667	*Lce1i*		
BB464727	*A830010M20Rik*			NM_010444	*Nr4a1*	+	+	NM_029688	*Srxn1*		
BB473548				NM_010500	*Ier5*	+		NM_053011	*Lrp1b*	+	
BB560177	*LOC620695*			NM_010516	*Cyr61*	+		NM_053182	*Pag1*		
BB667130	*2210038L17Rik*			NM_011012	*Oprl1*			NM_080433	*Zfp312*	+	
BB667296	*Ptprk*	+		NM_011111	*Serpinb2*	+		NM_080726	*Rem2*	+	
BC023116	*Cgref1*			NM_011198	*Ptgs2*	+		NM_080853	*Slc17a6*	+	
BE447663	*Heca*			NM_011245	*Rasgrf1*	+		NM_130447	*Dusp16*	+	
BE686667	*Slco5a1*			NM_011361	*Sgk*	+	+	NM_133236	*Glcci1*	+	
BE687858	*BC023892*			NM_011401	*Slc2a3*	+		NM_133753	*Errfi1*		
BE691546	*C030046G05*			NM_011607	*Tnc*	+	+	NM_133919	*Aff1*		
BE956940	*Lonrf3*			NM_011627	*Tpbg*	+		NM_144549	*Trib1*		
BG069873	*Gnb1l*	+		NM_011817	*Gadd45γ*	+	+	NM_144907	*Sesn2*	+	
BG071037	*BC049807*			NM_011982	*Homer1*	+		NM_145066	*Gpr85*	+	
BG917504	*Btaf1*			NM_011997	*Casp8ap2*	+		NM_145150	*Prc1*	+	
BM242340	*Pam*	+	+	NM_013498	*Crem*	+	+	NM_145463	*Tmem46*		
BM939903	*Fbxo33*			NM_013504	*Dsc1*	+		NM_145839	*Rasgef1b*		
BM941356	*Pcdh9*			NM_013562	*Ifrd1*	+	+	NM_145950	*Osgin2*		
BQ032894	*Thrap1*			NM_013598	*Kitl*	+		NM_153155	*C1ql3*		
BQ175781				NM_013613	*Nr4a2*	+	+	NM_153287	*Axud1*	+	
BQ176303	*9630037P07Rik*			NM_013628	*Pcsk1*	+		NM_153401	*Tieg3*	+	
BQ176417	*Cdh9*			NM_013642	*Dusp1*	+		NM_153553	*Npas4*		
M13227	*Penk1*			NM_013862	*Rabgap1l*			NM_172576	*Baz1a*		
NM_007498	*Atf3*	+	+	NM_015774	*Ero1l*	+		NM_175191	*Gpr22*	+	
NM_007540	*Bdnf*	+	+	NM_015828	*Gne*	+		NM_175312	*B630005N14Rik*	+	
NM_007553	*Bmp2*	+	+	NM_016789	*Nptx2*	+		NM_175341	*Mbnl2*	+	
NM_007570	*Btg2*	+	+	NM_016870	*Exod1*			NM_175428	*Zfp295*	+	
NM_007679	*Cebpd*	+		NM_017373	*Nfil3*	+	+	NM_175502	*Tmem74*		
NM_007681	*Cenpa*	+		NM_018790	*Arc*	+	+	NM_175513	*Zfp804a*		
NM_008005	*Fgf18*	+		NM_018820	*Sertad1*			NM_177075	*C030019I05Rik*	+	
NM_008013	*Fgl2*	+		NM_018869	*Gprk5*	+		NM_177468	*Spty2d1*		
NM_008017	*Smc2l1*	+		NM_019518	*Grasp*	+		NM_178892	*Tiparp*	+	
NM_008036	*Fosb*	+	+	NM_019927	*Arih1*			NM_181750	*R3hdm1*		
NM_008157	*Gpr19*	+		NM_019960	*Hspb3*	+		NM_183029	*Igf2bp2*		
NM_008327	*Ifi202b*	+		NM_019978	*Dcamkl1*	+		NM_199022	*Shc4*		
NM_008343	*Igfbp3*	+		NM_019986	*Habp4*	+		NM_201531	*Kcnf1*	+	
NM_008380	*Inhba*	+		NM_020265	*Dkk2*	+					
