# Supplementary material for: Nuclear Calcium Signaling Controls Expression of a Large Gene Pool: Identification of a Gene Program for Acquired Neuroprotection Induced by Synaptic Activity
Source: PLoS Genet. 2009 Aug 14;5(8):e1000604. doi: 10.1371/journal.pgen.1000604 (PMC2718706; doi:10.1371/journal.pgen.1000604)
Supplement: Text S1 — Supplemental Methods. (0.05 MB DOC) [file pgen.1000604.s003.doc]

**Quantitative reverse transcriptase PCR (QRT-PCR)**

To determine the relative mRNA expression levels of *c-fos*, *Bdnf*, *Atf3*, *Npas4*, *Ifi202b*, *Serpinb2*, *Inhba*, *Nr4a1*, *GADD45β*, *GADD45γ*, *Ptgs2*, *Btg2*, *Bcl6*, *Gusb*, and glyceraldehyde-3-phosphate dehydrogenase (*Gapdh*), QRT-PCR was performed using real-time TaqMan technology with a sequence detection system model 7300 Real Time PCR System (Applied Biosystems, Foster City, California, USA). Total RNA was extracted using RNeasy Plus Mini Kit (Qiagen GmbH, Germany) with additional on-column DNase I digestion during RNA purification. For the generation of the first stand cDNA, 3 to 5 µg of total RNA was reverse transcribed by extension of both random hexamer and oligo(dT)_20_ primers using SuperScript III reverse transcriptase (Invitrogen) according to the manufacturer’s instructions. QRT-PCR was performed using TaqMan Universal PCR Master Mix (Applied Biosystems) with Assays-on Demand^TM^ Gene Expression Products, which are ready to use primers and probe sets designed by Applied Biosystems. The mouse *c-fos*, *Bdnf*, *Atf3*, *Npas4*, *Ifi202b*, *Serpinb2*, *Inhba*, *Nr4a1*, *GADD45β*, *GADD45γ*, *Ptgs2*, and *Btg2*-specific probes were Assays-on-Demand^TM^ Gene Expression Products with TaqMan MGB probes, FAM dye-labelled (Assay ID Mm00476032 for *Atf3*, Mm00477633 for *Bcl6*, Mm00432069 for *Bdnf*, Mm00476162 for *Btg2*, Mm00435123 for *GADD45β*, Mm00487425 for *c-fos*, Mm00442225 for *GADD45γ*, Mm00463644 for *Npas4*, Mm00478374 for *Ptgs2*, Mm00839397 for *Ifi202b*, Mm00439358 for *Nr4a1*, Mm00440905 for *Serpinb2*, Mm00434338 for *Inhba* , Mm00446953 for *Gusb*, and Mm99999915 for *Gapdh*). The thermal cycling conditions comprised 10 min at 95°C, and 45 cycles of 15 sec for denaturation at 95°C and 60 sec for annealing and extension at 60°C. The expression level of the target mRNA was normalized to the relative ratio of the expression of *Gapdh* mRNA. Each QRT-PCR assay was performed at least three times, and the results are expressed as the mean ± SEM.

**Antibodies**

Antibodies to the following proteins were used: Flag (ANTI-FLAG M2 mouse monoclonal antibody; Sigma); hrGFP (rabbit polyclonal antibody; Stratagene); NeuN (mouse monoclonal antibody; Chemicon International, Inc.). Immunoblot and immunocytochemistry were done according to standard procedures.

**Recombinant adeno-associated virus (rAAV) and viral infections**

The mosaic serotypes rAAV1/2 were produced by co-transfection of human kidney cell line 293 (ATCC, Manassas, Virginia) by standard calcium phosphate precipitation. The 293 cells were grown in high-glucose-containing (4.5 g/liter) Dulbecco’s Modified Eagle Medium (DMEM; Life Technologies/Invitrogen, Carlsbad, CA) supplemented with 10% fetal bovine serum (Life Technologies/Invitrogen, Carlsbad, CA), 100 units/ml penicillin and 100 µg/ml streptomycin (Sigma, St. Louis, MO) at 37^o^C in a 5% humidified atmosphere. 2-3 hours before transfection, each 15-cm-diameter plate of 293 cells (70 to 80% confluent) was exchanged with 25 ml of fresh Iscove modified Dulbecco medium (IMDM; Life Technologies/Invitrogen, Carlsbad, CA) containing 5% fetal bovine serum without antibiotics. Packaging of chimeric serotypes rAAV1/2 transducing vectors was carried out with 25 µg of a mini-adenovirus helper plasmid pF∆6, 6.25 µg of AAV2 helper plasmid pRV1, 6.25 µg of AAV1 helper plasmid pH21 together with 12.5 µg of either rAAV-*LacZ,* rAAV-*CaMBP4*, rAAV-*CaMKIV(K75E)*, rAAV-*Atf3*, rAAV-*Npas4*, rAAV-*GADD45β*, rAAV-*GADD45γ*, rAAV-*Serpinb2*, rAAV-*Inhba*, rAAV-*Ifi202b*, rAAV-*Nr4a1*, rAAV-*Ptgs2*, rAAV-*Btg2*, rAAV-*Atf3**-*RNAi, rAAV-*Npas4-*RNAi, rAAV-*GADD45β-*RNAi, rAAV-*GADD45γ-*RNAi or rAAV-RNAi-Control for each 15-cm culture dish. 12 hours after transfection, the medium was replaced with fresh DMEM containing 10% fetal bovine serum and antibiotics. 60 hours after transfection, cells were harvested at low speed, resuspended in 100 mM NaCl-10 mM Tris-HCl (pH 8.5) and subjected to 3 cycles of freeze-thaw. The highly purified rAAV1/2 vector was purified using heparin affinity columns (HiTrap Heparin HP; GE Healthcare, Uppsala, Sweden). The rAAV1/2 vector stocks were concentrated in an Amicon Ultra-4 centrifugal filter devices 100K NMWL (Millipore, Bedford, MA). Genomic titers (genome copies/ml) of viral stocks were determined using a sequence detection system model 7300 Real Time PCR System (Applied Biosystems, Foster City, California, USA) with primers designed to woodchuck hepatitis virus post-translational regulatory element (WPRE) as described previously [1].

The vectors used to construct and package rAAVs have been described previously [1] and were provided by Matthias Klugmann and Matthew During. The rAAV protein expression cassette contains a cytomegalovirus enhancer (CMV)/chicken β-actin hybrid promoter (CBA), woodchuck hepatitis virus post-translational regulatory element (WPRE) and a bovine growth hormone polyA signal; for shRNA expression (i.e. rAAV-*Atf3-*RNAi, rAAV-*Npas4-*RNAi, rAAV-*GADD4β-*RNAi, rAAV-*GADD45γ-*RNAi or rAAV-RNAi-Control), an AAV vector was used that contains the U6 promoter for shRNA expression and a CMV/chicken β-actin hybrid promoter driving hrGFP expression. The following rAAVs were generated and confirmed by DNA sequencing: rAAV-*LacZ,* rAAV-*CaMBP4*, rAAV-*CaMKIV(K75E)*, rAAV-*Atf3*, rAAV-*Npas4*, rAAV-*GADD45β*, rAAV-*GADD45γ*, rAAV-*Serpinb2*, rAAV-*Inhba*, rAAV-*Ifi202b*, rAAV-*Nr4a1*, rAAV-*Ptgs2*, rAAV-*Btg2*, rAAV-*Atf3-*RNAi, rAAV-*Npas4-*RNAi, rAAV-*GADD45β-*RNAi, rAAV-*GADD45γ-*RNAi or rAAV-RNAi-Control. cDNA clones that served as templates for PCR-based cloning of the different rAAV constructs were provided by Dr. John Dedman (*CaMBP4*), Dr. Anthony Means (*CaMKIVK75E*) and Dr. Wieland Huttner (*Btg2*). Other clones were obtained using reverse-transcribed cDNA from mouse cultured hippocampal neurons. Hippocampal neurons were infected with recombinant rAAVs at 4 days *in vitro* (DIV). Infection efficiencies were routinely determined immunocytochemically at 9 DIV or 10 DIV using antibodies to the Flag tag or to hrGFP or by analyzing the fluorescence of hrGFP; they ranged from 80 to 95 percent of the viable neurons.

To construct rAAVs for expression of shRNA, oligonucleotides that contain the following sequences derived from the mouse *Atf3*, *Npas4*, *GADD45β* and *GADD45γ* were synthesized, annealed, and cloned into the *Bam*HI and *Hind*III sites of the rAAV vector:

5’-TCCTAGCCTGTCAACATAATA-3’ (AAV-*Atf3*-RNAi, sense)

5’-TATTATGTTGACAGGCTAGGA-3’ (AAV-*Atf3*-RNAi, anti-sense)

5’-TAGATGTGCTGTAGCTGCGAA-3’ (rAAV-*GADD45β*-RNAi, sense)

5’-TTCGCAGCTACAGCACATCTA-3’ (rAAV-*GADD45β*-RNAi, anti-sense)

5’-CGACTGCACTGCTCTTTCAAA-3’ (rAAV-*GADD45γ*-RNAi, sense)

5’-TTTGAAAGAGCAGTGCAGTCG-3’ (rAAV-*GADD45γ*-RNAi, anti-sense)

5’-TCTGTGACTTAACGTCTTCAA-3’ (rAAV-*Npas4*-RNAi, sense)

5’-TTGAAGACGTTAAGTCACAGA -3’ (rAAV-*Npas4*-RNAi, anti-sense)

5’-CGTCGCTTACCGATTCAGAAT-3’ (rAAV-Control-RNAi, sense)

5’-ATTCTGAATCGGTAAGCGACG-3’ (rAAV-Control-RNAi, anti-sense)

**Whole-genome transcription profiling**

Total cellular RNA was extracted from cultured mouse hippocampal neurons using RNeasy Mini kit (Qiagen GmbH, Hilden, Germany), and the contaminated DNA was removed with on-column DNase I digest by incubating the RNA with RNase-Free DNase I digest (Roche Applied Science, Mannheim, Germany). RNA quality was checked using RNA 6000 Nano LabChip Assay Kit on the Agilent 2100 Bioanalyzer (Agilent Technologies, Palo Alto, California, USA). A total of 8 µg of the RNA was converted to double-stranded cDNA using MessageAmp II aRNA Amplification Kit (Ambion, Texas, USA), and amplified cRNA was fragmented with fragmentation buffer [200 mM Tris-acetate (pH8.1), 500 mM KOAc and 150 mM MgOAc] and hybridized to the Affymetrix GeneChip Mouse Genome 430 2.0 array according to the manufacturer’s instructions, and the arrays were washed and stained on GeneChip Fluidics Station 450 and scanned with the Affymetrix 7G Scanner. Before each sample was hybridized to arrays, aliquots of each sample were hybridized to the Test3 array to verify the integrity of each labeled cRNA according to the manufacturer’s recommendations. A labeled cRNA was considered for subsequent analysis if the ratio of the average hybridization intensity of the 3’ end to that of the 5’ end of the glyceraldehyde-3-phosphate dehydrogenase gene (GAPDH) was less than 1.25. The gene chip experiments were done using three independently derived samples, i.e. for each of the three sets of gene chip experiments, hippocampal cultures were prepared, stimulated, and processed for analysis. Samples for the following conditions were generated: unstimulated control, bicuculline 4 hours, rAAV-*LacZ*, rAAV-*LacZ*/bicuculline 4 hours, rAAV-*CaMBP4*, rAAV-*CaMBP4*/bicuculline 4 hours. Prolonged expression of CaMBP4 following infection of hippocampal neurons on DIV 4 with rAAV-*CaMBP4* did not compromise the health of the neurons (analyzed on DIV 10 to 12) and did not compromise bicuculline-induced AP bursting and the generation of calcium transients (analyzed on DIV 10 to 12). The analysis of the gene chip data involved the use of two different programs, GCOS (Affymetrix, Santa Clara, CA, USA) and GeneSpring GX 7.3 (Agilent Technologies, Santa Clara, CA, USA); the raw data collected from the GeneChip Operating System were uploaded onto GeneSpring GX 7.3 for normalization purposes and additional analysis.

**Bioinformatics**

The data of Mouse 430 2.0 array are imported and normalized in GeneSpring software using the RMA algorithm [2]. Then, the analysis was performed in the (recommended) ‘Log of Ratio’ interpretation, in which the expression value for a condition is calculated as the geometric mean of all triplicates for that condition. In order to determine which probe sets are changed between two conditions, a fold induction or repression of at least 2 was required for indicating a probe set as either ‘up-regulated’ or ‘down-regulated’. In addition, a two-tailed Welch t-test (‘Parametric test, does not assume variances equal’) with a *p*-value cut-off of 0.05 was applied. No multiple testing corrections were done. All probe sets, which did not pass the t-test (*p*<0.05) or the fold change criteria, were considered ‘not changed’ between two conditions. A comprehensive list of all probe sets regulated by electrical activity is comprised of probe sets that, using the criteria described above, were ‘up-regulated’ or ‘down-regulated’ upon 4 hours of AP bursting. Nuclear calcium-regulated genes were selected based on the inhibitory effects of CaMBP4. For a regulatory event to be considered dependent on nuclear calcium signaling, all significant changes in expression caused by the stimulation must be reduced by at least 40% by CaMBP4. Furthermore, the expression values for with and without CaMBP4 must be found to be different with a *p*-value below 0.05 (according to a two-tailed Welch *t*-test). All filtering procedures were applied on individual probe set data. Annotations were retrieved from the Affymetrix website (CSV file of May 31, 2007). The GO Ontology Browser of GeneSpring was used to select all probe sets with GO term ‘apoptosis’ (GO 6915) from the list of nuclear calcium-regulated genes.

**Promoter analysis**

*In silico* promoter analysis was performed using consensus DNA binding sequence as described [3]. Briefly, genomic sequences were downloaded from the University of California Santa Cruz (UCSC) genome browser database for the most recent (mm9) assembly. The -5000 and +1000 regions relative to the transcriptional start sites were extracted and searched for the presence of full cAMP-response element (CRE) (TGACGTCA) or half CRE CGTCA site using custom Bioperl bases scripts generously provided by P.C. Boutros [4] (University of Toronto, Toronto, Canada).

**Assessment of apoptotic cells using Hoechst 33258 stain**

To assess apoptotic cell death, hippocampal neurons were stained with Hoechst 33258 and analyzed using fluorescence microscopy. Cells undergoing apoptosis showed shrunken nuclei (with loss of clearly identifiable nucleo-cytoplasmic borders) with several (often three or more) characteristic, large, nearly round, and brightly fluorescent chromatin clumps. Examples of Hoechst 33258 stained nuclei from apoptotic cells that have also been stained with TUNEL for comparison are shown in **Figure S1C**. In contrast, nuclei from healthy neurons showed clearly identifiable nucleo-cytoplasmic borders due to the light blue coloration of the entire nucleus and were decorated with more intensely fluorescent speckles. Examples of nuclei from healthy neurons stained with Hoechst 33258 are shown in **Figure S1B**.

**References**

1. Klugmann M, Symes CW, Leichtlein CB, Klaussner BK, Dunning J, et al. (2005) AAV-mediated hippocampal expression of short and long Homer 1 proteins differentially affect cognition and seizure activity in adult rats. Mol Cell Neurosci 28: 347-360.

2. Irizarry RA, Bolstad BM, Collin F, Cope LM, Hobbs B, et al. (2003) Summaries of Affymetrix GeneChip probe level data. Nucleic Acids Res 31: e15.

3. Zhang X, Odom DT, Koo SH, Conkright MD, Canettieri G, et al. (2005) Genome-wide analysis of cAMP-response element binding protein occupancy, phosphorylation, and target gene activation in human tissues. Proc Natl Acad Sci U S A 102: 4459-4464.

4. Pastorelli R, Carpi D, Campagna R, Airoldi L, Pohjanvirta R, et al. (2006) Differential expression profiling of the hepatic proteome in a rat model of dioxin resistance: correlation with genomic and transcriptomic analyses. Mol Cell Proteomics 5: 882-894.
